# The S2–S3 Loop of Kv7.4 Channels Is Essential for Calmodulin Regulation of Channel Activation

**DOI:** 10.3389/fphys.2020.604134

**Published:** 2021-01-20

**Authors:** Wenhui Zhuang, Zhiqiang Yan

**Affiliations:** ^1^State Key Laboratory of Medical Neurobiology and MOE Frontiers Center for Brain Science, Department of Physiology and Biophysics, School of Life Sciences, Human Phenome Institute, Fudan University, Shanghai, China; ^2^Shenzhen Bay Laboratory, Institute of Molecular Physiology, Shenzhen, China

**Keywords:** Kv7.4 channels, calmodulin regulation, calcium-dependent regulation, channel activation, voltage-dependent activation

## Abstract

Kv7.4 (KCNQ4) voltage-gated potassium channels control excitability in the inner ear and the central auditory pathway. Mutations in Kv7.4 channels result in inherited progressive deafness in humans. Calmodulin (CaM) is crucial for regulating Kv7 channels, but how CaM affects Kv7 activity has remained unclear. Here, based on electrophysiological recordings, we report that the third EF hand (EF3) of CaM controls the calcium-dependent regulation of Kv7.4 activation and that the S2–S3 loop of Kv7.4 is essential for the regulation mediated by CaM. Overexpression of the mutant CaM_1234_, which loses the calcium binding ability of all four EF hands, facilitates Kv7.4 activation by accelerating activation kinetics and shifting the voltage dependence of activation leftwards. The single mutant CaM_3_, which loses the calcium binding ability of the EF3, phenocopies facilitating effects of CaM_1234_ on Kv7.4 activation. Kv7.4 channels co-expressed with wild-type (WT) CaM show inhibited activation when intracellular calcium levels increase, while Kv7.4 channels co-expressed with CaM_1234_ or CaM_3_ are insensitive to calcium. Mutations C156A, C157A, C158V, R159, and R161A, which are located within the Kv7.4 S2–S3 loop, dramatically facilitate activation of Kv7.4 channels co-expressed with WT CaM but have no effect on activation of Kv7.4 channels co-expressed with CaM_3_, indicating that these five mutations decrease the inhibitory effect of Ca^2+^/CaM. The double mutation C156A/R159A decreases Ca^2+^/CaM binding and completely abolishes CaM-mediated calcium-dependent regulation of Kv7.4 activation. Taken together, our results provide mechanistic insights into CaM regulation of Kv7.4 activation and highlight the crucial role of the Kv7.4 S2–S3 loop in CaM regulation.

## Introduction

Kv7 (KCNQ) voltage-gated potassium channels (Kv7.1–7.5; Gutman et al., 2003) produce Iks current (Kv7.1; Barhanin et al., 1996; Sanguinetti et al., 1996; Wang et al., 1996; Jentsch, 2000) and M current (Kv7.2–7.5; Wang et al., 1998; Delmas and Brown, 2005; Hernandez et al., 2008), playing critical roles in controlling cellular excitability in the brain, heart, and ear. Kv7 channels open at subthreshold membrane potentials and function as a brake on membrane excitation (Delmas and Brown, 2005; Brown and Passmore, 2009). Kv7.4 channels are expressed in the inner ear and the central auditory pathway in the brainstem (Kubisch et al., 1999; Beisel et al., 2000, 2005; Kharkovets et al., 2000). Mutations in Kv7.4 result in an autosomal-dominant, non-syndromic, progressive, high-frequency hearing loss, denoted as DFNA2 (Kubisch et al., 1999).

Calmodulin (CaM) appears to be an essential auxiliary subunit of Kv7 channels and strongly modulates Kv7 function. The Kv7 A and B helices, which are located in the cytoplasmic C-terminal domain and close to the pore, are identified as CaM binding sites (Wen and Levitan, 2002; Yus-Najera et al., 2002; Sachyani et al., 2014; Strulovich et al., 2016; Sun and MacKinnon, 2017; Chang et al., 2018). Disrupted CaM interactions with Kv7.1 (Ghosh et al., 2006; Shamgar et al., 2006), Kv7.2 (Etxeberria et al., 2008; Alaimo et al., 2009, 2018), heteromeric Kv7.2/Kv7.3 (Liu and Devaux, 2014), or Kv7.4 (Chang et al., 2018) have been shown to be tightly associated with severely impaired Kv7 surface expression, which illustrates the vital role of CaM in Kv7 assembly and trafficking. Numerous studies about the effects of calcium on the interaction between CaM and Kv7 revealed that both Ca^2+^/CaM and Apo/CaM forms bind to the channels (Wen and Levitan, 2002; Yus-Najera et al., 2002; Ghosh et al., 2006; Shamgar et al., 2006; Bal et al., 2008), whereas details of the structure of the Kv7:CaM complex are calcium dependent. Apo/CaM embraces an antiparallel pair of the Kv7 A and B helices, with the Apo/C-lobe interacting with the A helix and the Apo/N-lobe interacting with the B helix (Bernardo-Seisdedos et al., 2018; Chang et al., 2018). Calcium binding allows the Ca^2+^/N-lobe to stay anchored on the Kv7 B helix in a similar configuration to the apo/N-lobe (Sachyani et al., 2014; Strulovich et al., 2016; Sun and MacKinnon, 2017; Bernardo-Seisdedos et al., 2018; Chang et al., 2018), while there are striking differences between the Ca^2+^/C-lobe and Apo/C-lobe conformations. Structural studies of the interaction between CaM and the Kv7.4 A and B helices showed that calcium binding makes the C-lobe lose interaction with the A helix and bind to the B helix weakly through a much smaller interaction surface (Xu et al., 2013; Chang et al., 2018). CaM acts as a calcium sensor and regulates Kv7 currents in a calcium-dependent manner. Ca^2+^/CaM facilitates Kv7.1 function (Sachyani et al., 2014; Tobelaim et al., 2017; Chang et al., 2018) but inhabits Kv7.2–7.5 (Gamper and Shapiro, 2003; Gamper et al., 2005; Sihn et al., 2016; Gomis-Perez et al., 2017; Chang et al., 2018). Nevertheless, the exact mechanism by which CaM regulates Kv7 channels remains unclear.

The cryo-EM structure of the Kv7.1/CaM complex reports a newly discovered interaction between the third EF hand (EF3) of CaM and the Kv7.1 S2–S3 loop (Sun and MacKinnon, 2017), which is unique to Kv7 channels but absent in the other voltage-gated potassium channels (Supplementary Figure S1), raising a possibility that CaM contacts the voltage sensor of Kv7 channels through the S2–S3 loop. In our study, using electrophysiological recordings and co-immunoprecipitation assays, we demonstrate that CaM regulates Kv7.4 activation through the S2–S3 loop and propose a new model accounting for CaM-mediated regulation of Kv7 channels.

## Materials and Methods

### Plasmid Constructs

The plasmids (EX-U0210-M98-5) expressing human Kv7.4 (NP_004691.2) were purchased from GeneCopoeia. Each amino acid of the Kv7.4 S2–S3 loop was substituted by an alanine or a valine to generate Kv7.4 mutants. DNA encoding CaM (NP_008819.1) was a gift from Jiahuai Han (Xiamen University, China). CaM mutants with impaired calcium affinities had an alanine substitution of the first residue, an aspartate, and in the calcium-binding loop of each EF hand (CaM_3_, D94A; CaM_4_, D130A; CaM_12_, D21A/D57A; CaM_34_, D94A/D130A; and CaM_1234_, D21A/D57A/D94A/D130A; Geiser et al., 1991; Xia et al., 1998). Mutations G97A, N98A, G99A, and Y100A were introduced into wild-type (WT) CaM (referred to as CaM G97A, CaM N98A, CaM G99A, and CaM Y100A) or CaM_3_ (referred to as CaM_3_ G97A, CaM_3_ N98A, CaM_3_ G99A, and CaM_3_ Y100A). All mutants were generated by homologous recombination and verified by automated sequencing. DNA segments encoding WT or mutant Kv7.4 were subcloned into pIRES2-EGFP plasmids, and segments encoding WT or mutant CaM were subcloned into pIRES2-mCherry plasmids.

There are two alternatively spliced isoforms of human Kv7.4, called Kv7.4 isoform a and Kv7.4 isoform b (NP_751895.1). Most studies including ours used Kv7.4 isoform a, which we and others called Kv7.4. Our study also performed experiments on Kv7.4 isoform b, which we called Kv7.4b to distinguish from isoform a. DNA segments encoding Kv7.4b were generated by deleting residues 377–430 of Kv7.4 isoform a by homologous recombination and subcloned into pIRES2-EGFP plasmids. The sequence was verified by automated sequencing.

For surface labeling, the Kv7.4 subunit was tagged with a modified Myc epitope that was flanked with the extracellular D1–D2 loop of ClC-5 chloride channels to increase accessibility in the extracellular loop that connects the transmembrane domains S1 and S2 as previously described (Kim et al., 2011; referred to as Myc-Kv7.4). The amino acid sequence of the Kv7.4 S1–S2 loop was changed to STIQEHQELANENSEHEQKLISEEDLVTFEERDKCPEWNC. The Myc-Kv7.4 segments were subcloned into pcDNA3.1 plasmids without fluorescent tags. DNA encoding CD8α was kindly provided by Lan Bao (the Chinese Academy of Sciences, China), and an HA epitope was inserted between the signal peptide and the mature protein of CD8α (referred to as HA-CD8α). The HA-CD8α segments were subcloned into pcDNA3.1 plasmids as well.

For measurements of Kv7.4 and CaM total expressions and co-immunoprecipitation experiments, the Kv7.4 subunit was tagged with a Flag epitope (DYKDDDDK) at the N-terminus (referred to as Flag-Kv7.4), and CaM was tagged with an HA epitope (YPYDVPDYA) at the C-terminus (referred to as CaM-HA).

### Electrophysiology

Chinese hamster ovary (CHO) cells were obtained from the Cell Bank of the Chinese Academy of Sciences, and cultured in DMEM/F-12 (Gibco) with 10% fetal bovine serum (FBS) at 37°C with 5% CO_2_. Cells in 35 mm diameter wells were transfected using Lipofectamine 3000 (Invitrogen) with 1 μg of DNA encoding Kv7.4 channels and 1 μg of either DNA encoding CaM or empty vectors per dish.

Twenty-four hours following transfection, the media were replaced with bath solution. The bath solution contained 145 mM NaCl, 4 mM KCl, 1.8 mM CaCl_2_, 0.5 mM MgCl_2_, 10 mM HEPES, and 5 mM D-glucose (pH 7.4 adjusted with NaOH). Electrophysiology experiments were performed at room temperature by whole-cell voltage clamp recordings using polished pipettes filled with pipette solution. The pipettes were pulled from borosilicate glass capillaries (BF150-86-10, Sutter Instrument, Novato) using a Flaming/Brown micropipette puller (P-97, Sutter Instrument, Novato) and polished to obtain 3–5 MΩ resistance. The normal pipette solution contained 140 mM KCl, 1 mM MgCl_2_, 10 mM HEPES, 10 mM EGTA, 3 mM CaCl_2_, and 4 mM K_2_ATP (pH 7.2 adjusted with KOH).

Pipette solutions containing various free calcium concentrations were used to change intracellular calcium levels. Whole-cell recordings were performed on transfected cells using pipette solutions containing 140 mM KCl, 1 mM MgCl_2_, 10 mM HEPES, 4 mM K_2_ATP, and 10 mM EGTA plus 0, 3, or 6 mM CaCl_2_. The free calcium concentrations of the pipette solutions were 0, 77.6, and 275.9 nM, respectively, as calculated by the Webmax software online.^1^

BAPTA-AM (Abcam) was used to decrease intracellular calcium levels. BAPTA-AM was prepared as a stock solution in 20 mM in DMSO. One microliter of the BAPTA-AM stock solution was added to 1 ml of DMEM/F-12 media without FBS to obtain a final concentration of 20 μM BAPTA-AM. Twenty-four hours following transfection, cells were pretreated with the media containing 20 μM BAPTA-AM for 6 h at 37°C with 5% CO_2_. Whole-cell recordings were performed on transfected cells pretreated with BAPTA-AM using the pipette solution containing 140 mM KCl, 1 mM MgCl_2_, 10 mM HEPES, 4 mM K_2_ATP, and 10 mM EGTA (pH 7.2 adjusted with KOH).

All recordings were carried out using pClamp10.5 software [(Axon Instruments, United States), an Axopatch 200B amplifier (Axon Instruments, United States), and an Axon Digidata 1550 digitizer (Axon Instruments, United States)]. Data were filtered at 1 kHz and digitized at 10 kHz. Kv7.4 channel current traces were recorded from a holding potential of −80 mV to depolarizing step potentials ranging from −100 to 40 mV with 20 mV increments for 1,000 ms, followed by −50 mV pulses for 500 ms to generate tail currents. Currents were measured after at least 80% series resistance compensation. Leakage currents were digitally subtracted after P/N leak subtraction. Kv7.4 current amplitudes at varying test potentials were measured at steady-state levels, and the currents were then divided by the cell capacitance (pF) to generate a current density-voltage relationship. Tail currents were measured immediately after pulsing to −50 mV. To obtain voltage-dependent activation curves, the normalized tail current vs. voltage was plotted and fitted with the following Boltzmann equation: I/Imax = 1/{1 + exp[(V_1/2_ − Vm)/k]}, where V_1/2_ is the half-activation potential, Vm is the membrane potential, and k is the slope factor. Parameters of Kv7.4 activation kinetics were determined by fitting the activation phase of the traces with a two-component exponential function: I(t) = A_1_ exp (−t/*τ*_1_) + A_2_ exp (−t/τ_2_) + C, where I is the recorded current, A is the current amplitude, τ is the time constant, and C is the amplitude at which the activation starts. A_1_, τ_1_ and A_2_, τ_2_ represent the parameters for the fast and slow activation components, respectively. The deactivation time constants were measured by fitting the deactivating phase to a single exponential at −50 mV, as described above.

### Immunofluorescence and Quantification of Surface Expression

Chinese hamster ovary cells in 35 mm diameter wells were transfected with 1 μg of DNA encoding Myc-tagged Kv7.4 channels, 0.3 μg of DNA encoding HA-tagged CD8α, and 1 μg of either DNA encoding CaM or empty vectors per dish. Forty-eight hours after co-transfection with Myc-Kv7.4, HA-CD8α, and CaM, live CHO cells were placed in phosphate-buffered saline (PBS) with 3% bovine serum albumin (BSA) for 1 h to block unspecific binding. The cells were then incubated for 30 min with primary antibodies, mouse monoclonal anti-Myc (30601ES20, Yeasen), and rabbit monoclonal anti-HA (ab236632, Abcam). After three washes with PBS, the cells were incubated for 30 min with the secondary antibodies, Alexa-594-conjugated donkey anti-mouse IgG (H + L; 34112ES60, Yeasen), and Alexa-488-conjugated donkey anti-rabbit IgG (H + L; 34206ES60, Yeasen). All procedures were performed at room temperature.

Confocal images were acquired on a laser scanning confocal microscope (FV1200, Olympus, Japan) using a 60× oil immersion objective, in multitracking mode to minimize channel crosstalk. The surface expression of Kv7.4 channels was quantified by the ratio of Myc fluorescence intensity to HA fluorescence intensity of all the cells captured in random subsets, and fluorescence intensities were measured using ImageJ (National Institutes of Health, United States). HA-CD8α was used here as a control for its stable and exclusive expression on the cell surface (Zhang et al., 2008).

### Co-immunoprecipitation and Western Blot

Chinese hamster ovary cells in 100 mm diameter wells were transfected with 9 μg of DNA encoding Flag-tagged Kv7.4 channels and 9 μg of DNA encoding HA-tagged CaM per dish. Forty-eight hours after transfection, cells were lysed in lysis buffer containing 50 mM Tris-HCl (pH 8), 150 mM NaCl, 0.5% Nonidet P-40, 200 μM Na_3_VO_4_, and protease inhibitor cocktail (B14001, Bimake), rotating at 4°C for 30 min. Cell lysates were centrifuged at 14,000 *g* for 10 min at 4°C. Collected supernatants were incubated with rabbit monoclonal anti-Flag antibodies (ab205606, Abcam) in the presence of either 2 mM CaCl_2_ or 2 mM EGTA overnight at 4°C. Protein A + G agarose beads (P2012, Beyotime) were added, and the mixture was incubated for additional 3 h at 4°C. The beads that pulled down the immune complexes were collected by centrifugation at 1,000 *g* for 2 min, and then washed three times with lysis buffer. SDS-PAGE loading buffer was added and boiled for 5 min to elute proteins. Immunoprecipitated proteins were separated by SDS-PAGE and blotted onto nitrocellulose membranes (10600001, GE Healthcare). The blots were blocked in TBST (10 mM Tris-HCl, 150 mM NaCl, and 0.1% Tween 20, pH 7.5) with 5% nonfat milk at room temperature for 1 h, and then incubated with mouse anti-Flag antibodies (30502ES20, Yeasen) and mouse anti-HA antibodies (30701ES20, Yeasen) overnight. After three washes with TBST, the blots were incubated with horseradish peroxidase (HRP)-labeled goat anti-mouse IgG (H + L; 33201ES60, Yeasen) for 1 h at room temperature. Proteins were visualized using Immun-Star HRP Substrate (1705041, Bio-Rad) and ChemiDoc Imaging System (Bio-Rad, United States). Chemiluminescent signals were collected by Image Lab software (Bio-Rad, United States) and analyzed by ImageJ (National Institutes of Health, United States).

### Statistics

The statistical significances were determined by unpaired Student’s *t*-test for comparisons between two groups and by one-way ANOVA for comparisons of more groups. All statistical data in the figures were presented as mean ± SEM.

## Results

### CaM Regulates Kv7.4 Activation in a Calcium-Dependent Manner

**Figure 1:**
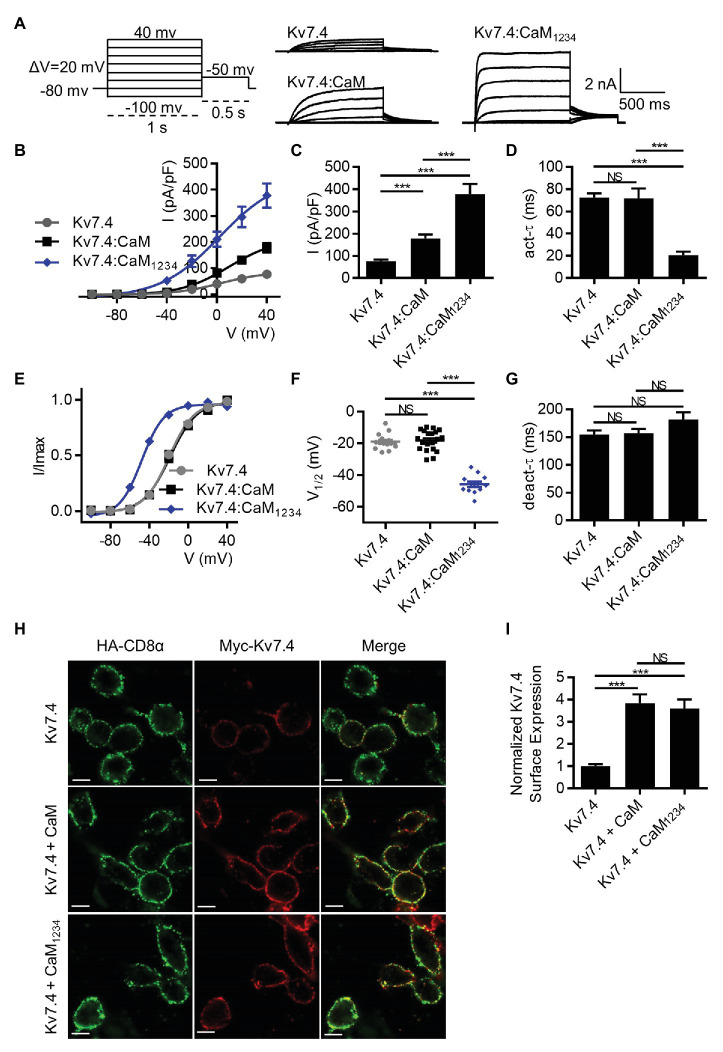
Calmodulin (CaM) regulates Kv7.4 activation and trafficking. **(A)** Representative current traces of Kv7.4 channels without overexpression of exogenous CaM and Kv7.4 channels with overexpression of exogenous CaM or CaM_1234_ using the indicated protocol. **(B)** Corresponding current density-voltage relations of Kv7.4 (*N* = 20), Kv7.4:CaM (*N* = 20), and Kv7.4:CaM_1234_ (*N* = 12). **(C)** Average current densities at 40 mV for Kv7.4 (*N* = 20), Kv7.4:CaM (*N* = 20), and Kv7.4:CaM_1234_ (*N* = 12). **(D)** Average time constants at 40 mV for the fast activation component of Kv7.4 (*N* = 20), Kv7.4:CaM (*N* = 20), and Kv7.4:CaM_1234_ (*N* = 12). **(E)** Voltage-dependent activation curves for Kv7.4 (*N* = 20), Kv7.4:CaM (*N* = 20), and Kv7.4:CaM_1234_ (*N* = 12). **(F)** Individual V_1/2_ values for Kv7.4 (*N* = 20), Kv7.4:CaM (*N* = 20), and Kv7.4:CaM_1234_ (*N* = 12). **(G)** Average time constants at 40 mV for the deactivation of Kv7.4 (*N* = 20), Kv7.4:CaM (*N* = 20), and Kv7.4:CaM_1234_ (*N* = 12). **(H)** Immunofluorescence analysis of surface expression of Kv7.4 channels without overexpression of exogenous CaM and Kv7.4 channels with overexpression of exogenous CaM or CaM_1234_. Live transfected cells were double labeled with anti-HA (green) and anti-Myc (red) antibodies to detect surface expression of HA-CD8α and Myc-Kv7.4, respectively. The scale bars indicate 10 μm. **(I)** Quantification of surface expression of K7.4 channels without overexpression of exogenous CaM (*n* = 30) and Kv7.4 channels with overexpression of exogenous CaM (*n* = 30) or CaM_1234_ (*n* = 30), in which the ratio (Myc intensity/HA intensity) of each subset was normalized to the average ratio of Kv7.4 without overexpression of exogenous CaM. *n* indicates the number of captured subsets obtained from five replications of experiments. Error bars show SEM. *N* indicates the number of cells. Asterisks indicate significance: ^**^p < 0.01; ^***^p < 0.001. *NS* indicates not significant.

Previous studies reported CaM conferred calcium sensitivity to Kv7.4 channels and affected Kv7.4 activity (Sihn et al., 2016; Chang et al., 2018). To test the calcium-dependent regulation mediated by CaM, we first measured currents of CHO cells transfected with Kv7.4 alone or together with WT CaM or the mutant CaM_1234_ that lost the calcium binding ability of all four EF hands. CHO cells have endogenous CaM (Xu et al., 2007), and overexpression of endogenous WT CaM or CaM_1234_ could knock down the effects of endogenous CaM. Shown in Figure 1A are whole-cell current traces of Kv7.4 channels with only endogenous CaM but no overexpressed exogenous CaM and Kv7.4 channels with overexpression of exogenous WT CaM or CaM_1234_. Compared with Kv7.4 channels with only endogenous CaM of CHO cells, overexpression of exogenous WT CaM and CaM_1234_ significantly increased current amplitudes, and Kv7.4 co-expressed with CaM_1234_ generated the largest current amplitudes (Figures 1B,C; current densities at 40 mV: Kv7.4 = 76.7 ± 6.6 pA/pF; Kv7.4:CaM = 178.2 ± 17.9 pA/pF; and Kv7.4:CaM_1234_ = 377.6 ± 35.9 pA/pF). Kv7.4 with overexpression of exogenous WT CaM showed activation properties similar to Kv7.4 channels with only endogenous CaM in terms of activation kinetics (Figure 1D; *τ* values at 40 mV: Kv7.4 = 72.4 ± 3.7 ms; Kv7.4:CaM = 72.0 ± 8.6 ms) and the voltage dependence of activation (Figures 1E,F; V_1/2_ values: Kv7.4 = −19.9 ± 0.8 mV; Kv7.4:CaM = −18.8 ± 1.6 mV), while overexpression of exogenous CaM_1234_ sharply accelerated the activation rate (Figure 1D; *τ* value at 40 mV: Kv7.4:CaM_1234_ = 20.8 ± 3.1 ms) and shifted the half-activation voltage leftward markedly (Figures 1E,F; V_1/2_ value: Kv7.4:CaM_1234_ = −47.9 ± 1.0 mV). Overexpression of exogenous WT CaM or CaM_1234_ did not change deactivation kinetics (Figure 1G).

We also measured the surface expression of Kv7.4 without overexpression of exogenous CaM and Kv7.4 with overexpression of exogenous WT CaM or CaM_1234_ to further interpret the different current amplitudes. To quantify surface expression, we developed a new method based on immunofluorescence assays. CHO cells were co-transfected with Myc-Kv7.4, HA-CD8α and empty vectors, WT CaM or CaM_1234_. The Kv7.4 subunit was tagged with a Myc epitope in the extracellular loop that connects the transmembrane domains S1 and S2, which has been used to detect Kv7.4 surface expression in previous studies (Kim et al., 2011). Our electrophysiological data showed that the extracellular Myc tag had no effects on Kv7.4 currents or CaM regulation of Kv7.4 (Supplementary Figure S2). An extracellular HA tag was inserted between the signal peptide and the mature protein of CD8α. Previous studies have shown that CD8α could be stably and exclusively present on the cell surface (Zhang et al., 2008). Hence, HA-CD8α was used as an indicator to observe the expression of exogenous protein and as a ruler to quantify the membrane area of the transfected cells. The fluorescence intensity of Myc-Kv7.4 was divided by the fluorescence intensity of the co-expressed HA-CD8α, and this ratio could represent an average surface expression of Kv7.4 channels. Statistically, overexpression of exogenous WT CaM or CaM_1234_ significantly increased Kv7.4 surface expression about 3.5-fold (Figures 1H,I). Therefore, overexpression of exogenous WT CaM increased Kv7.4 current amplitudes by enhancing Kv7.4 surface expression without changing activation properties, while overexpression of exogenous CaM_1234_ increased Kv7.4 current amplitudes by enhancing Kv7.4 surface expression as well as facilitating channel activation. There was no significant difference in surface expression between Kv7.4 co-expressed with WT CaM and Kv7.4 co-expressed with CaM_1234_ (Figures 1H,I), which emphasized that only facilitated activation of Kv7.4 co-expressed with CaM_1234_ accounted for its greater current amplitudes than Kv7.4 co-expressed with WT CaM. Thus, we focused on the effects of WT or mutant CaM on Kv7.4 activation in the subsequent study.

**Figure 2:**
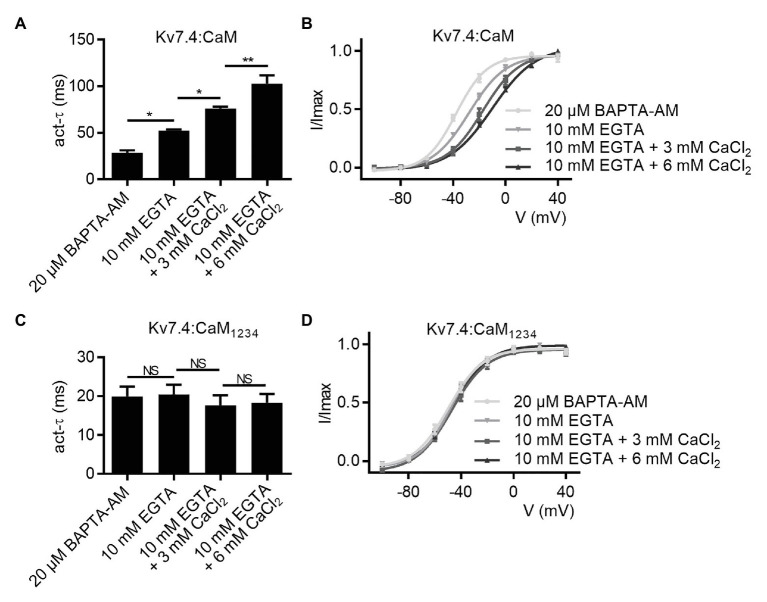
CaM mediates calcium-dependent inhibition to Kv7.4 activation. **(A)** Average time constants at 40 mV for the fast activation component of Kv7.4:CaM with pipette solutions containing only 10 mM EGTA (*N* = 7), 10 mM EGTA plus 3 mM CaCl_2_ (*N* = 7), or 10 mM EGTA plus 6 mM CaCl_2_ (*N* = 7) and Kv7.4:CaM pretreated with BAPTA-AM (*N* = 8; **B**) Voltage-dependent activation curves for Kv7.4:CaM with pipette solutions containing only 10 mM EGTA (*N* = 7), 10 mM EGTA plus 3 mM CaCl_2_ (*N* = 7), or 10 mM EGTA plus 6 mM CaCl_2_ (*N* = 7) and Kv7.4:CaM pretreated with BAPTA-AM (*N* = 8). **(C)** Average time constants at 40 mV for the fast activation component of Kv7.4:CaM_1234_ with pipette solutions containing only 10 mM EGTA (*N* = 7), 10 mM EGTA plus 3 mM CaCl_2_ (*N* = 7), or 10 mM EGTA plus 6 mM CaCl_2_ (*N* = 7) and Kv7.4:CaM pretreated with BAPTA-AM (*N* = 9) **(D)** Voltage-dependent activation curves for Kv7.4:CaM_1234_ with pipette solutions containing only 10 mM EGTA (*N* = 7), 10 mM EGTA plus 3 mM CaCl_2_ (*N* = 7), or 10 mM EGTA plus 6 mM CaCl_2_ (*N* = 7) and Kv7.4:CaM pretreated with BAPTA-AM (*N* = 9). Error bars show SEM. *N* indicates the number of cells. Asterisks indicate significance: ^*^p < 0.05; ^**^p < 0.01. *NS* indicates not significant.

We next sought to verify the role of calcium in CaM regulation of Kv7.4 activation to explain the differences in activation properties between Kv7.4 co-expressed with WT CaM and Kv7.4 co-expressed with CaM_1234_. We prepared different pipette solutions containing 10 mM EGTA plus 0, 3, or 6 mM CaCl_2_, and the free calcium concentrations are calculated by the MaxChelator software to be about 0, 80, and 270 nM, respectively. We performed whole-cell recordings using the different pipette solutions to change intracellular calcium levels of the recorded cells. Increasing free calcium concentrations in the pipette solutions exerted inhibitory effects on Kv7.4:CaM activation, decreasing the activation rate (Figure 2A; *τ* values at 40 mV: Kv7.4:CaM with 10 mM EGTA = 52.3 ± 1.2 ms; Kv7.4:CaM with 10 mM EGTA plus 3 mM CaCl_2_ = 76.0 ± 2.0 ms; Kv7.4:CaM with 10 mM EGTA plus 6 mM CaCl_2_ = 102.4 ± 9.2 ms), and shifting the voltage-dependent activation rightwards (Figure 2B; V_1/2_ values at 40 mV: Kv7.4:CaM with 10 mM EGTA = −27.8 ± 1.4 mV; Kv7.4:CaM with 10 mM EGTA plus 3 mM CaCl_2_ = −17.3 ± 1.6 mV; Kv7.4:CaM with 10 mM EGTA plus 6 mM CaCl_2_ = −10.1 ± 1.1 mV). However, both activation kinetics (Figure 2C; *τ* values at 40 mV: Kv7.4:CaM_1234_ with 10 mM EGTA = 20.5 ± 2.5 ms; Kv7.4:CaM_1234_ with 10 mM EGTA plus 3 mM CaCl_2_ = 17.7 ± 2.6 ms; Kv7.4:CaM_1234_ with 10 mM EGTA plus 6 mM CaCl_2_ = 18.4 ± 2.2 ms) and voltage-dependent activation (Figure 2B; V_1/2_ values at 40 mV: Kv7.4:CaM_1234_ with 10 mM EGTA = −48.0 ± 1.4 mV; Kv7.4:CaM_1234_ with 10 mM EGTA plus 3 mM CaCl_2_ = −47.2 ± 1.8 mV; Kv7.4:CaM_1234_ with 10 mM EGTA plus 6 mM CaCl_2_ = −45.7 ± 1.1 mV) of Kv7.4:CaM_1234_ remained unchanged when we used different pipette solutions. Therefore, we further confirmed that functional CaM mediated calcium-dependent inhibition to Kv7.4 channel activation and that the calcium-insensitive CaM_1234_ facilitated Kv7.4 activation essentially by abolishing the calcium-dependent inhibition.

Compared with the pipette solutions with CaCl_2_ added, the pipette solution containing only 10 mM EGTA facilitated Kv7.4:CaM activation. Nevertheless, there are still differences between Kv7.4:CaM with the pipette solution containing only 10 mM EGTA and Kv7.4:CaM_1234_ in both the activation rate and the voltage dependence of activation, possibly because binding to Kv7.4 made CaM obtain high affinities for calcium *in vivo* and the calcium chelating ability of 10 mM EGTA was not enough to make all CaM in Apo/CaM forms, which CaM_1234_ mimicked, in such a limited time of the whole-cell recording. We pretreated transfected cells with 20 μM BAPTA-AM, a cell permeable calcium chelator, for 6 h and performed whole-cell recordings using the pipette solution containing only 10 mM EGTA. Pretreating with BAPTA-AM further facilitated Kv7.4:CaM activation but had no effect on Kv7.4:CaM_1234_ activation in terms of the activation rate (Figures 2A,C; *τ* values at 40 mV: Kv7.4:CaM pretreated with 20 μM BAPTA-AM = 28.4 ± 2.8 ms; Kv7.4:CaM_1234_ pretreated with 20 μM BAPTA-AM = 19.9 ± 2.5 ms) and the voltage dependence of activation (Figures 2B,D; V_1/2_ values: Kv7.4:CaM pretreated with 20 μM BAPTA-AM = −37.6 ± 1.0 mV; Kv7.4:CaM2_234_ pretreated with 20 μM BAPTA-AM = −49.8 ± 1.7 mV).

Taken together, our results proved that CaM regulates Kv7.4 channels in a calcium-dependent manner, in which Apo/CaM facilitated Kv7.4 activation and Ca^2+^/CaM inhibited Kv7.4 activation.

### The CaM EF3 Is Vital to the Regulation of Kv7.4 Activation

**Figure 3:**
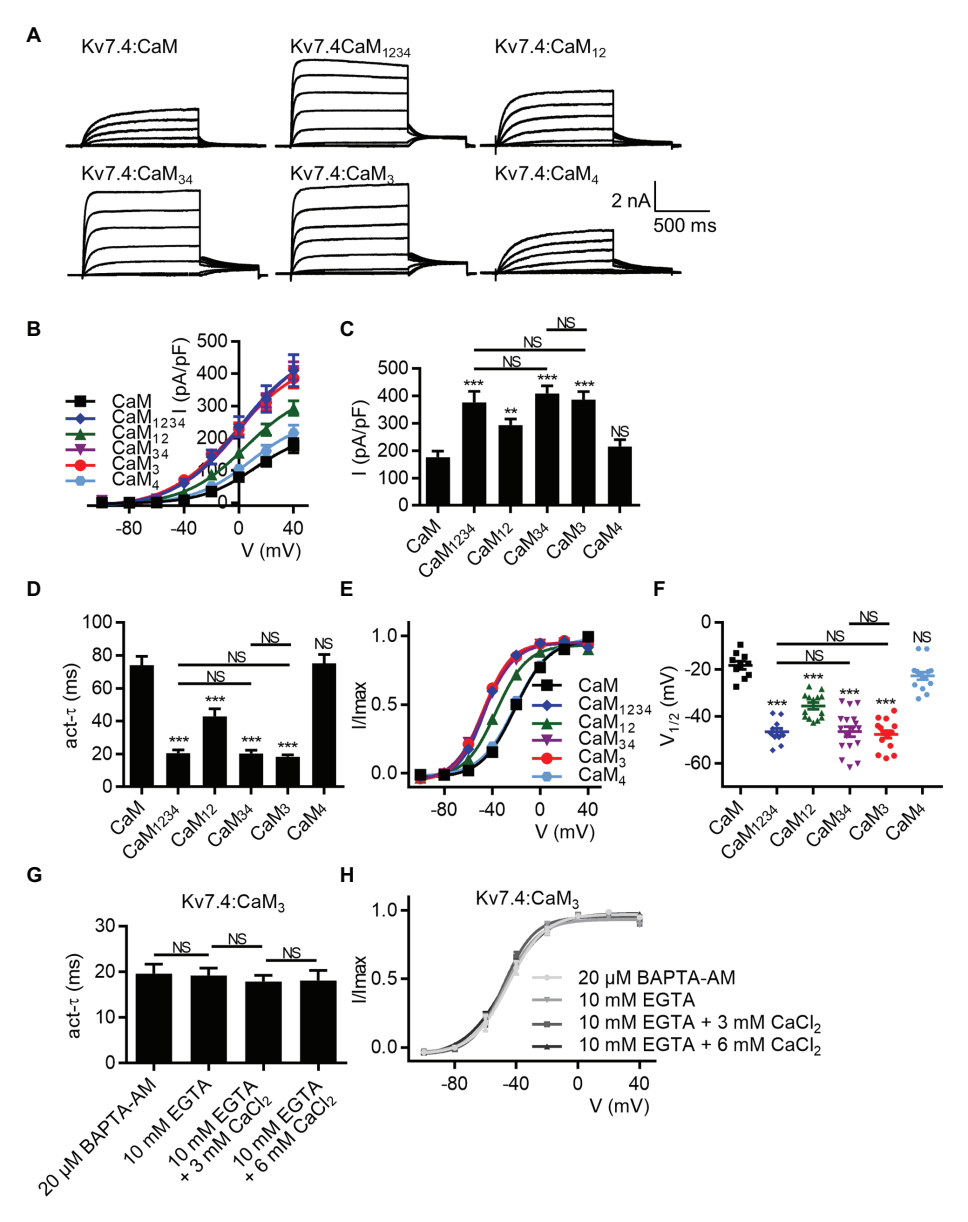
The CaM EF3 controls the regulation of Kv7.4 activation. **(A)** Representative current traces of cells co-expressing Kv7.4 channels with CaM or CaM mutants. **(B)** Corresponding current density-voltage relations of Kv7.4 channels with CaM (*N* = 10) or CaM mutants: CaM_1234_ (*N* = 11), CaM_12_ (*N* = 14), CaM_34_ (*N* = 17), CaM_3_ (*N* = 15), and CaM_4_ (*N* = 14). **(C)** Average current densities at 40 mV for Kv7.4 channels with CaM (*N* = 10) or CaM mutants: CaM_1234_ (*N* = 11), CaM_12_ (*N* = 14), CaM_34_ (*N* = 17), CaM_3_ (*N* = 15), and CaM_4_ (*N* = 14). **(D)** Average time constants at 40 mV for the fast activation component of Kv7.4 with CaM (*N* = 10) or CaM mutants: CaM_1234_ (*N* = 11), CaM_12_ (*N* = 14), CaM_34_ (*N* = 17), CaM_3_ (*N* = 15), and CaM_4_ (*N* = 14). **(E)** Voltage-dependent activation curves for Kv7.4 with CaM (*N* = 10) or CaM mutants: CaM_1234_ (*N* = 11), CaM_12_ (*N* = 14), CaM_34_ (*N* = 17), CaM_3_ (*N* = 15), and CaM_4_ (*N* = 14). **(F)** Individual V_1/2_ values for Kv7.4 with CaM (*N* = 10) or CaM mutants: CaM_1234_ (*N* = 11), CaM_12_ (*N* = 14), CaM_34_ (*N* = 17), CaM_3_ (*N* = 15), and CaM_4_ (*N* = 14). **(G)** Average time constants at 40 mV for the fast activation component of Kv7.4:CaM_3_ with pipette solutions containing only 10 mM EGTA (*N* = 8), 10 mM EGTA plus 3 mM CaCl_2_ (*N* = 7), or 10 mM EGTA plus 6 mM CaCl_2_ (*N* = 8) and Kv7.4:CaM_3_ pretreated with BAPTA-AM (*N* = 9). **(H)** Voltage-dependent activation curves for Kv7.4:CaM_3_ with pipette solutions containing only 10 mM EGTA (*N* = 8), 10 mM EGTA plus 3 mM CaCl_2_ (*N* = 7), or 10 mM EGTA plus 6 mM CaCl_2_ (*N* = 8) and Kv7.4:CaM_3_ pretreated with BAPTA-AM (*N* = 9). Error bars show SEM. *N* indicates the number of cells. Asterisks indicate significance: ^**^p < 0.01; ^***^p < 0.001. *NS* indicates not significant.

Earlier studies have reported calcium-dependent structural properties of the Kv7.4:CaM complex, in which calcium binding to the CaM N-lobe or C-lobe led to different interactions between CaM and Kv7.4 (Xu et al., 2013; Chang et al., 2018). To test if the calcium-dependent structural properties had effects on the calcium-dependent regulation of Kv7.4 activation mediated by CaM, we sought to explore the role of each lobe or EF hand of CaM in regulating Kv7.4 channels. Hence, we co-expressed Kv7.4 with the CaM mutants that lost the calcium binding affinity of the N-lobe (CaM_12_) or the C-lobe (CaM_34_), and the effects of CaM_12_ and CaM_34_ were compared with the effects of WT CaM and CaM_1234_. There was no significant difference among the expression levels of WT CaM and the CaM mutants (Supplementary Figure S4). Shown in Figure 3A are whole-cell current traces of Kv7.4 channels co-expressed with WT CaM and the CaM mutants. CaM_34_ mirrored the effects of CaM_1234_ on the current amplitudes (Figures 3B,C; current densities at 40 mV: Kv7.4:CaM_1234_ = 375.7 ± 40.8 pA/pF; Kv7.4:CaM_34_ = 408.9 ± 28.0 pA/pF), the activation rate (Figure 3D; *τ* values at 40 mV: Kv7.4:CaM_1234_ = 20.6 ± 2.0 ms; Kv7.4:CaM_34_ = 20.4 ± 2.0 ms), and the voltage dependence (Figures 3E,F; V_1/2_ values: Kv7.4:CaM_34_ = −46.3 ± 1.0 mV; CaM_34_ = −47.1 ± 1.2 mV). Compared with CaM_1234_ and CaM_34_, CaM_12_ caused much smaller changes to the indicated Kv7.4 channel properties, only generating a small increase in the current amplitudes (Figures 3B,C; current densities at 40 mV: Kv7.4:CaM_12_ = 293.5 ± 22.2 pA/pF), a mildly faster activation rate (Figure 3D; *τ* values at 40 mV: Kv7.4:CaM_12_ = 42.9 ± 4.7 ms), and a slightly leftward shift in the voltage-dependent activation curve (Figures 3E,F; V_1/2_ values: Kv7.4:CaM_12_ = −36.9 ± 1.4 mV). These results suggested that the CaM C-lobe played a key role in the calcium-dependent regulation of Kv7.4 activation, although calcium binding to the N-lobe also conferred a slight inhibition to Kv7.4.

Next, we used the CaM mutants with disabled calcium binding ability of the EF3 (CaM_3_) or the EF4 (CaM_4_) in the C-lobe. CaM_3_ phenocopied the effects of CaM_1234_ and CaM_34_ on current amplitudes (Figures 3B,C; current densities at 40 mV: Kv7.4:CaM_3_ = 385.8 ± 30.3 pA/pF), activation kinetics (Figure 3D; *τ* values at 40 mV: Kv7.4:CaM_3_ = 18.5 ± 1.0 ms), and voltage-dependent activation (Figures 3E,F; V_1/2_ values: Kv7.4:CaM_3_ = −48.0 ± 0.9 mV). However, CaM_4_, which caused effects almost equivalent to WT CaM, made no difference to the current amplitudes (Figures 3B,C; current densities at 40 mV: Kv7.4:CaM_4_ = 216.0 ± 24.2 pA/pF), the activation rate (Figure 3D; *τ* values at 40 mV: Kv7.4:CaM_4_ = 75.2 ± 5.4 ms), or the voltage dependence of activation (Figures 3E,F; V_1/2_ values: Kv7.4:CaM_4_ = −22.2 ± 1.2 mV). Like Kv7.4:CaM_1234_, Kv7.4:CaM_3_ showed insensitivity to the changing intracellular calcium levels, and both varying free calcium concentrations of the pipette solutions and application of BAPTA-AM have no effect on the activation rate (Figure 3G; *τ* values at 40 mV: Kv7.4:CaM_3_ pretreated with 20 μM BAPTA-AM = 19.6 ± 2.1 ms; Kv7.4:CaM_3_ with 10 mM EGTA = 19.2 ± 1.6 ms; Kv7.4:CaM_3_ with 10 mM EGTA plus 3 mM CaCl_2_ = 17.9 ± 1.4 ms; Kv7.4:CaM_3_ with 10 mM EGTA plus 6 mM CaCl_2_ = 18.1 ± 2.2 ms) or the voltage-dependent activation (Figure 3H; V_1/2_ values: Kv7.4:CaM_3_ pretreated with 20 μM BAPTA-AM = −45.3 ± 1.5 mV; Kv7.4:CaM_3_ with 10 mM EGTA = −47.3 ± 1.7 mV; Kv7.4:CaM_3_ with 10 mM EGTA plus 3 mM CaCl_2_ = −48.5 ± 1.2 mV; Kv7.4:CaM_3_ with 10 mM EGTA plus 6 mM CaCl_2_ = −47.1 ± 1.3 mV) of Kv7.4:CaM_3_. These results emphasized a critical role of the CaM EF3 in modulating Kv7.4 activation, which supported the possibility that the CaM EF3 interacted with the Kv7.4 S2–S3 loop to contact the voltage sensor (Sun and MacKinnon, 2017).

### The Kv7.4 S2–S3 Loop Is Required for CaM Regulation of Kv7.4 Activation

**Figure 4:**
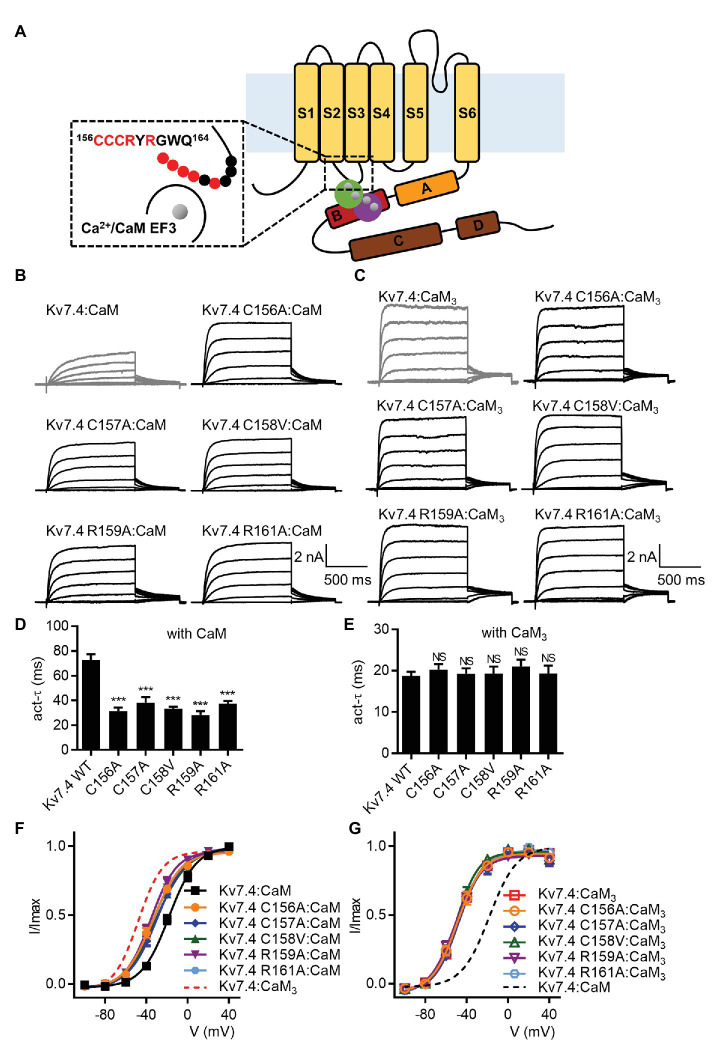
Mutations in the Kv7.4 S2-S3 loop affect CaM regulation of channel activation. **(A)** A schematic depicting a possible structural model of Ca^2+^/CaM interacting with the Kv7.4 subunit, in which both the Ca^2+^/N-lobe and Ca^2+^/C-lobe interact with the B helix of Kv7.4 channels, based on structural studies of Ca^2+^/CaM with the Kv7.4 A and B helices (Xu et al., 2013; Chang et al., 2018). A magnified view of the potential interface between the Kv7.4 S2–S3 loop and the Ca^2+^/CaM EF3 is shown in the dashed box. The nine residues of the Kv7.4 S2–S3 loop studied in this work are labeled with circles. Red circles indicate residues required for CaM regulation of Kv7.4 activation (according to our electrophysiological). **(B)** Representative current traces of cells co-expressing Kv7.4 WT or Kv7.4 mutants with CaM. **(C)** Representative current traces of cells co-expressing Kv7.4 WT or Kv7.4 mutants with CaM_3_. **(D)** Average time constants at 40 mV for the fast activation component of Kv7.4:CaM (*N* = 12), Kv7.4 C156A:CaM (*N* = 13), Kv7.4 C157A:CaM (*N* = 8), Kv7.4 C158V:CaM (*N* = 10), Kv7.4 R159A:CaM (*N* = 9), and Kv7.4 R161A:CaM (*N* = 13). **(E)** Average time constants at 40 mV for the fast activation component of Kv7.4:CaM_3_ (*N* = 11), Kv7.4 C156A:CaM_3_ (*N* = 11), Kv7.4 C157A:CaM_3_ (*N* = 10), Kv7.4 C158V:CaM_3_ (*N* = 9), Kv7.4 R159A:CaM_3_ (*N* = 8), and Kv7.4 R161A:CaM_3_ (*N* = 9). **(F)** Voltage-dependent activation curves for Kv7.4:CaM (*N* = 12), Kv7.4 C156A:CaM (*N* = 13), Kv7.4 C157A:CaM (*N* = 8), Kv7.4 C158V:CaM (*N* = 10), Kv7.4 R159A:CaM (*N* = 9), and Kv7.4 R161A:CaM (*N* = 13). **(G)** Voltage-dependent activation curves for Kv7.4:CaM_3_ (*N* = 11), Kv7.4 C156A:CaM_3_ (*N* = 11), Kv7.4 C157A:CaM_3_ (*N* = 10), Kv7.4 C158V:CaM_3_ (*N* = 9), Kv7.4 R159A:CaM_3_ (*N* = 8), and Kv7.4 R161A:CaM_3_ (*N* = 9). Error bars show SEM. *N* indicates the number of cells. Asterisks indicate significance: ^***^p < 0.001. *NS* indicates not significant.

We sought to interpret the importance of the CaM EF3 in regulating Kv7.4 activation based on the potential interaction between the CaM EF3 and the Kv7 S2–S3 loop (Sun and MacKinnon, 2017). Each residue of the nine-amino-acid S2–S3 loop was changed to an alanine or a valine. Because the current traces from CHO cells expressing C158A or G162A were quite noisy (Supplementary Figure S6A), we generated the mutants C158V and G162V for further investigation. When co-expressed with WT CaM, five of the nine Kv7.4 mutants displayed facilitated channel activation (Figure 4A) with a faster activation rate (Figure 4D; *τ* values at 40 mV: Kv7.4:CaM = 72.7 ± 4.8 ms, Kv7.4 C156A:CaM = 31.3 ± 2.9 ms, Kv7.4 C157A:CaM = 38.2 ± 4.6 ms, Kv7.4 C158V:CaM = 33.3 ± 1.7 ms, Kv7.4 R159A:CaM = 28.3 ± 3.0 ms, and Kv7.4 R161A:CaM = 37.4 ± 2.1 ms) and a leftward shift in the voltage-dependent activation (Figure 4F; V_1/2_ values: Kv7.4:CaM = −18.2 ± 1.1 mV, Kv7.4 C156A:CaM = −33.9 ± 1.2 mV, Kv7.4 C157A:CaM = −29.6 ± 1.1 mV, Kv7.4 C158V:CaM = −30.6 ± 1.2 mV, Kv7.4 R159A:CaM = −35.7 ± 1.2 mV, and Kv7.4 R161A:CaM = −33.0 ± 1.1 mV). However, these five mutants co-expressed CaM_3_ showed activation properties similar to WT Kv7.4 co-expressed with CaM_3_ in terms of both the activation kinetics (Figure 4E; *τ* values at 40 mV: Kv7.4:CaM_3_ = 18.8 ± 1.0 ms, Kv7.4 C156A: Kv7.4:CaM_3_ = 20.2 ± 1.4 ms, Kv7.4 C157A:CaM_3_ = 19.2 ± 1.4 ms, Kv7.4 C158V:CaM_3_ = 19.3 ± 1.8 ms, Kv7.4 R159A:CaM_3_ = 21.0 ± 1.7 ms, and Kv7.4 R161A:CaM_3_ = 19.3 ± 2.0 ms) and the voltage dependence of activation (Figure 4G; V_1/2_ values: Kv7.4:CaM_3_ = −47.9 ± 0.8 mV, Kv7.4 C156A:CaM_3_ = −49.1 ± 1.5 mV, Kv7.4 C157A:CaM_3_ = −48.1 ± 1.4 mV, Kv7.4 C158V:CaM_3_ = −50.1 ± 1.3 mV, Kv7.4 R159A:CaM_3_ = −51.2 ± 1.5 mV, and Kv7.4 R161A:CaM_3_ = −49.6 ± 0.9 mV), indicating that these five mutations had no effect on activation of Kv7.4 co-expressed with CaM_3_. These results suggested that mutations within the S2–S3 loop of Kv7.4 channels decreased the calcium-dependent inhibition mediated by functional CaM (Supplementary Figure S7) and proved that the S2–S3 loop of Kv7.4 is required for CaM regulation of Kv7.4 activation. We could not probe the role of the residues Tyr160 and Gly162 in CaM regulation, because the mutants Y160A and G162V did not yield functional currents (Supplementary Figures S6B, S8A,B). Moreover, the mutations W163A and Q164A did not affect the currents of Kv7.4 co-expressed with WT CaM (Supplementary Figures S8C,E) or CaM_3_ (Supplementary Figures S8D,F), implying that the two residues were not involved in CaM regulation.

### The Kv7.4 Double Mutation C156A/R159A Completely Abolishes CaM Regulation of Kv7.4 Activation

**Figure 5:**
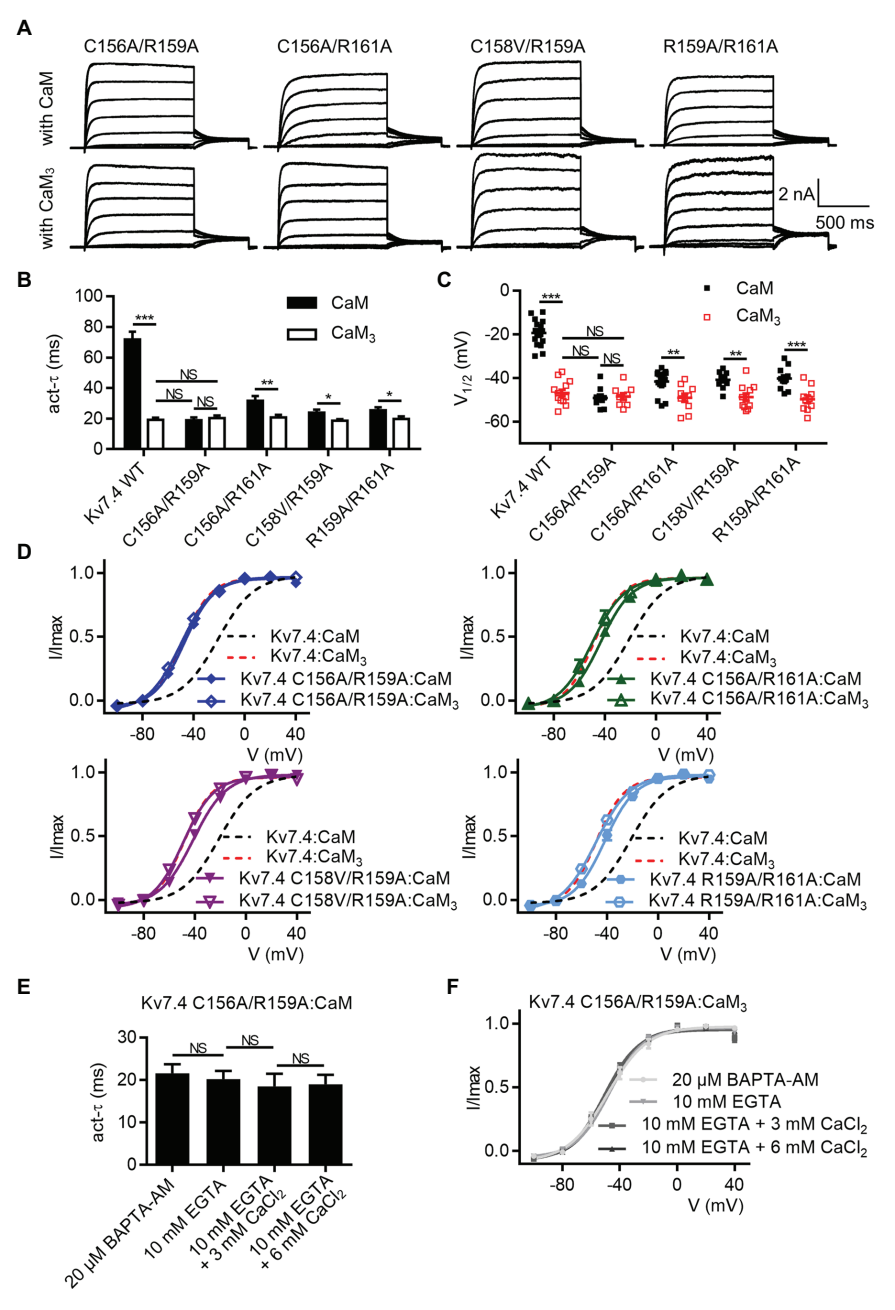
The Kv7.4 double mutation C156A/R159A completely abolishes CaM regulation of channel activation. **(A)** Representative current traces of cells co-expressing Kv7.4 double mutants with CaM (top) or CaM_3_ (bottom). **(B)** Average time constants at 40 mV for the fast activation component of Kv7.4:CaM (*N* = 17), Kv7.4:CaM_3_ (*N* = 13), Kv7.4 C156A/R159A:CaM (*N* = 9), Kv7.4 C156A/R159A:CaM_3_ (*N* = 9), Kv7.4 C156A/R161A:CaM (*N* = 17), Kv7.4 C156A/R161A:CaM_3_ (*N* = 10), Kv7.4 C158V/R159A:CaM (*N* = 10), Kv7.4 C158V/R159A:CaM_3_ (*N* = 11), Kv7.4 R159A/R161A:CaM (*N* = 11), and Kv7.4 R159A/R161A:CaM_3_ (*N* = 10). **(C)** Individual V_1/2_ values for Kv7.4:CaM (*N* = 17), Kv7.4:CaM_3_ (*N* = 13), Kv7.4 C156A/R159A:CaM (*N* = 9), Kv7.4 C156A/R159A:CaM_3_ (*N* = 9), Kv7.4 C156A/R161A:CaM (*N* = 17), Kv7.4 C156A/R161A:CaM_3_ (*N* = 10), Kv7.4 C158V/R159A:CaM (*N* = 10), Kv7.4 C158V/R159A:CaM_3_ (*N* = 11), Kv7.4 R159A/R161A:CaM (*N* = 11), and Kv7.4 R159A/R161A:CaM_3_ (*N* = 10). **(D)** Voltage-dependent activation curves for Kv7.4:CaM (*N* = 17), Kv7.4:CaM_3_ (*N* = 13), Kv7.4 C156A/R159A:CaM (*N* = 9), Kv7.4 C156A/R159A:CaM_3_ (*N* = 9), Kv7.4 C156A/R161A:CaM (*N* = 17), Kv7.4 C156A/R161A:CaM_3_ (*N* = 10), Kv7.4 C158V/R159A:CaM (*N* = 10), Kv7.4 C158V/R159A:CaM_3_ (*N* = 11), Kv7.4 R159A/R161A:CaM (*N* = 11), and Kv7.4 R159A/R161A:CaM_3_ (*N* = 10). **(E)** Average time constants at 40 mV for the fast activation component of Kv7.4 C156A/R159A:CaM with pipette solutions containing only 10 mM EGTA (*N* = 7), 10 mM EGTA plus 3 mM CaCl_2_ (*N* = 7), or 10 mM EGTA plus 6 mM CaCl_2_ (*N* = 7) and Kv7.4 C156A/R159A:CaM pretreated with BAPTA-AM (*N* = 8). **(F)** Voltage-dependent activation curves for Kv7.4 C156A/R159A:CaM with pipette solutions containing only 10 mM EGTA (*N* = 7), 10 mM EGTA plus 3 mM CaCl_2_ (*N* = 7), or 10 mM EGTA plus 6 mM CaCl_2_ (*N* = 7) and Kv7.4 C156A/R159A:CaM pretreated with BAPTA-AM (*N* = 8). *N* indicates the number of cells. Asterisks indicate significance: ^*^p < 0.05; ^**^p < 0.01; ^***^p < 0.001. *NS* indicates not significant.

Although the five Kv7.4 mutations (C156A, C157A, C158V, R159A, and R161A) facilitated activation of Kv7.4 channels co-expressed with WT CaM, the activation time constants and the half-activation voltages of the five single mutants were still different from the parameters of Kv7.4 channels co-expressed with CaM_3_, suggesting that the inhibitory effects of Ca^2+^/CaM was decreased but not abolished completely. For further investigation on the necessity of the S2–S3 loop, we sought to enhance the facilitating effects by generating double mutants (C156A/R159A, C156A/R161A, C158V/R159A, and R159A/R158A). Shown in Figure 5A are whole-cell current traces of the double mutants co-expressed with WT CaM or CaM_3_. When co-expressed with WT CaM, all the double mutants displayed further facilitated channel activation in terms of activation kinetics (Figure 5B; *τ* values at 40 mV: Kv7.4:CaM = 71.9 ± 5.0 ms, Kv7.4 C156A/R159A:CaM = 19.1 ± 1.7 ms, Kv7.4 C156A/R161A:CaM = 31.7 ± 3.1 ms, Kv7.4 C158V/R159A:CaM = 23.9 ± 2.0 ms, and Kv7.4 R159A/R161A:CaM = 25.5 ± 1.9 ms) and voltage-dependent activation (Figures 5C,D; V_1/2_ values: Kv7.4:CaM = −21.1 ± 1.2 mV, Kv7.4 C156A/R159A = −47.3 ± 1.2 mV, Kv7.4 C156A/R161A:CaM = −43.3 ± 0.7 mV, Kv7.4 C158V/R159A:CaM = −41.3 ± 0.8 mV, and Kv7.4 C158V/R159A:CaM = −41.7 ± 1.1 mV). As expected, all the double mutants co-expressed with CaM_3_ generated activation rates (Figure 4B; *τ* values at 40 mV: Kv7.4:CaM_3_ = 19.2 ± 1.4 ms, Kv7.4 C156A/R159A:CaM_3_ = 20.5 ± 1.6 ms, Kv7.4 C156A/R161A:CaM_3_ = 20.8 ± 1.7 ms, Kv7.4 C158V/R159A:CaM_3_ = 18.6 ± 1.1 ms, and Kv7.4 R159A/R161A:CaM_3_ = 19.9 ± 1.6 ms) and half-activation voltages (Figures 4C,D; V_1/2_ values: Kv7.4:CaM_3_ = −48.6 ± 0.9 mV, Kv7.4 C156A/R159A: CaM_3_ = −49.9 ± 0.8 mV, Kv7.4 C156A/R161A:CaM_3_ = −50.0 ± 1.8 mV, Kv7.4 C158V/R159A:CaM_3_ = −49.0 ± 0.9 mV, and Kv7.4 R159A/R161A:CaM_3_ = −48.8 ± 1.8 mV; Figures 4C,D) similar to WT Kv7.4 channels with CaM_3_. Importantly, according to our statistical results, there was no significant difference among Kv7.4 C156A/R159A:CaM, Kv7.4 C156A/R159A:CaM_3_, and Kv7.4:CaM_3_ in both activation kinetics (Figure 5B) and voltage-dependent activation (Figure 5C). This indicated that the double mutation C156A/R159A generated facilitating effects equivalent to CaM_3_ and suggested that CaM regulation of Kv7.4 activation was abolished completely by this mutation. Additionally, both varying free calcium concentrations of the pipette solutions and application of BAPTA-AM have no effect on activation kinetics (Figure 5E; *τ* values at 40 mV: Kv7.4 C156A/R159A:CaM pretreated with 20 μM BAPTA-AM = 21.6 ± 2.1 ms; Kv7.4 C156A/R159A:CaM with 10 mM EGTA = 20.2 ± 1.9 ms; Kv7.4 C156A/R159A:CaM with 10 mM EGTA plus 3 mM CaCl2 = 18.5 ± 3.0 ms; Kv7.4 C156A/R159A:CaM with 10 mM EGTA plus 6 mM CaCl2 = 19.0 ± 2.3 ms) or voltage-dependent activation (Figure 5F; V1/2 values: Kv7.4 C156A/R159A:CaM pretreated with 20 μM BAPTA-AM = −48.6 ± 1.7 mV; Kv7.4 C156A/R159A:CaM with 10 mM EGTA = −47.2 ± 1.3 mV; Kv7.4 C156A/R159A:CaM with 10 mM EGTA plus 3 mM CaCl2 = −50.9 ± 1.4 mV; Kv7.4 C156A/R159A:CaM with 10 mM EGTA plus 6 mM CaCl2 = −48.6 ± 1.1 mV) of Kv7.4 C156A/R159A:CaM. Taken together, these results of Kv7.4 C156A/R159A further emphasized the essential role of the Kv7.4 S2–S3 loop in CaM-mediated calcium-dependent regulation.

**Figure 6:**
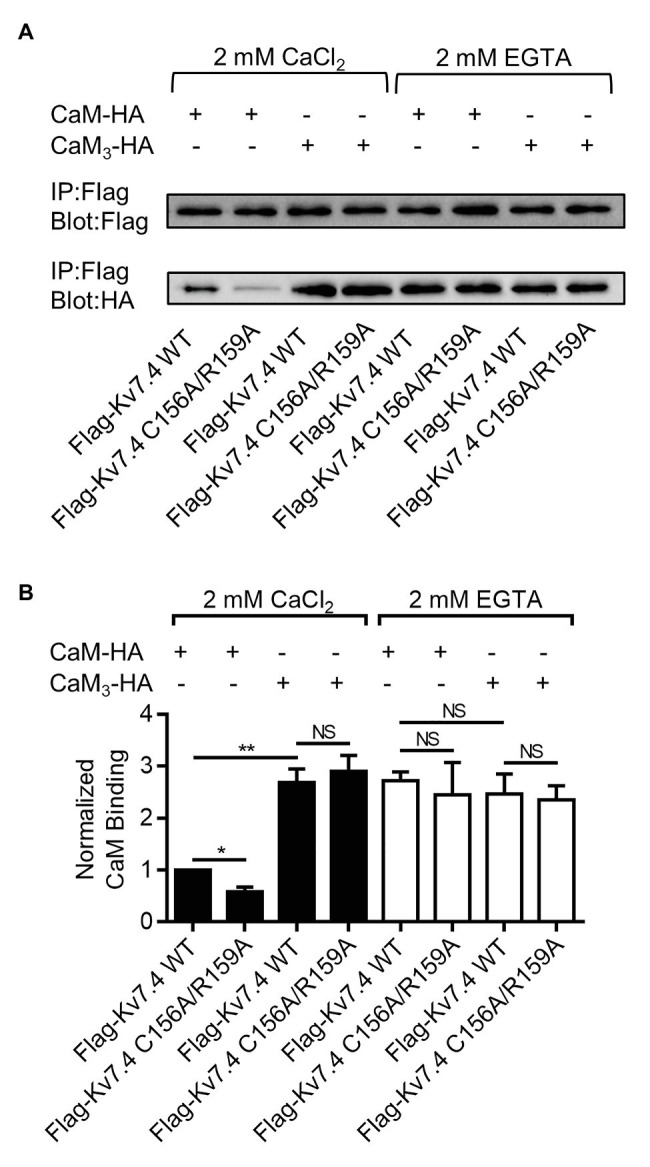
The Kv7.4 double mutation C156A/R159A decreased Ca^2+^/CaM binding. **(A)** Co-immunoprecipitation analysis of CaM or CaM_3_ binding to Kv7.4 or Kv7.4 C156A/R159A. Chinese hamster ovary (CHO) cells were co-transfected with Flag-Kv7.4 and CaM-HA, Flag Kv7.4 C156A/R159A and CaM-HA, Flag-Kv7.4 and CaM_3_-HA, or Flag-Kv7.4 C156A/R159A:CaM_3_-HA. Immunoprecipitation was accomplished using anti-Flag antibodies in the presence of either 2 mM CaCl_2_ or 2 mM EGTA. The immunoprecipitates were probed with anti-Flag antibodies to confirm precipitation of Flag-Kv7.4 or Flag-Kv7.4 C156A/R159A and were probed with anti-HA antibodies to detect CaM-HA or CaM_3_-HA pulled down with the channels. The experiments were replicated four times and showed similar results. **(B)** Quantification of CaM or CaM_3_ binding to Kv7.4 or Kv7.4 C156A/R159A. The binding efficiency is represented as the ratio of HA intensity to Flag intensity, and all ratios are normalized to the ratio representing CaM binding to Kv7.4 in the presence of CaCl_2_. Asterisks indicate significance: ^*^p < 0.05; ^**^p < 0.01. *NS* indicates not significant.

We used co-immunoprecipitation experiments to probe the effects of the double mutation C156/R159A, which completely abolished CaM regulation of Kv7.4 activation, on Kv7.4 forming complex with CaM, a requirement for their interaction. A Flag tag and an HA tag were introduced to the N-terminus of Kv7.4 and the C-terminus of CaM, respectively. Electrophysiological recordings revealed no noticeable distinction between tagged and untagged combinations of Kv7.4 with CaM in channel currents (Supplementary Figure S3A) or in the effects of CaM regulation (Supplementary Figures S3B,C). Immunoprecipitation accomplished using anti-Flag antibodies to precipitate Flag-Kv7.4 or Flag-Kv7.4 C156A/R159A in the presence of either 2 mM CaCl2 or 2 mM EGTA. The immunoprecipitates were probed with anti-Flag antibodies to confirm precipitation of Flag-Kv7.4 or Flag-Kv7.4 C156A/R159A and were probed with anti-HA antibodies to detect CaM-HA or CaM_3_-HA pulled down with the channels. In the presence of 2 mM CaCl_2_, WT CaM showed a detectable but much weaker association with the Kv7.4 C156A/R159A mutant than with WT Kv7.4, while the signals of CaM_3_ pulled down with WT Kv7.4 and Kv7.4 C156A/R159A are similar (Figure 6). These results implied that the Kv7.4 S2-S3 loop interacted with Ca^2+^/CaM but had no association with Ca^2+^/CaM_3_ that had an empty EF3 and calcium-bound EF hands 1, 2, and 4. Of note, the signal of CaM_3_ pulled down with Kv7.4 channels was stronger than WT CaM in the presence of 2 mM CaCl_2_, matching the observation by Kosenko and Hoshi (2013), who reported that longer exposure to calcium decreased CaM binding to Kv7.2 channels. This difference might result from the different interactions of the Kv7.4 C-terminal domain with Ca^2+^/CaM and with CaM_3_, in which calcium binding disturbed the interaction between the CaM C-lobe and the Kv7.4 A helix and the Ca^2+^/C-lobe bound to the B helix weakly through a much smaller interaction surface (Xu et al., 2013; Chang et al., 2018). Therefore, the stronger binding of CaM_3_ than Ca^2+^/CaM is not contradictory to our hypothesis that Ca^2+^/CaM interacted with the Kv7.4 S2–S3 loop, whereas Ca^2+^/CaM_3_ lost this interaction. In the presence of 2 mM EGTA through the whole immunoprecipitation overnight, both WT CaM and CaM_3_ would exist in Apo/CaM forms, and there were no significant differences among the signals of WT CaM pulled down with WT Kv7.4, WT CaM pulled down with Kv7.4 C156A/R159A, CaM_3_ pulled down with WT Kv7.4, and CaM_3_ pulled down with Kv7.4 C156A/R159A (Figure 6). Additionally, the binding of Apo/CaM is stronger than Ca^2+^/CaM and similar to Ca^2+^/CaM_3_, suggesting an important role of the EF3 in CaM binding. Taken together, these results of the co-immunoprecipitation experiments coincided with our electrophysiological data, supporting an potential correlation between the structure and function of the Kv7.4:CaM complex.

### The Residues N98 and N99 in the CaM EF3 Are Required for the Regulation of Kv7.4 Activation

**Figure 7:**
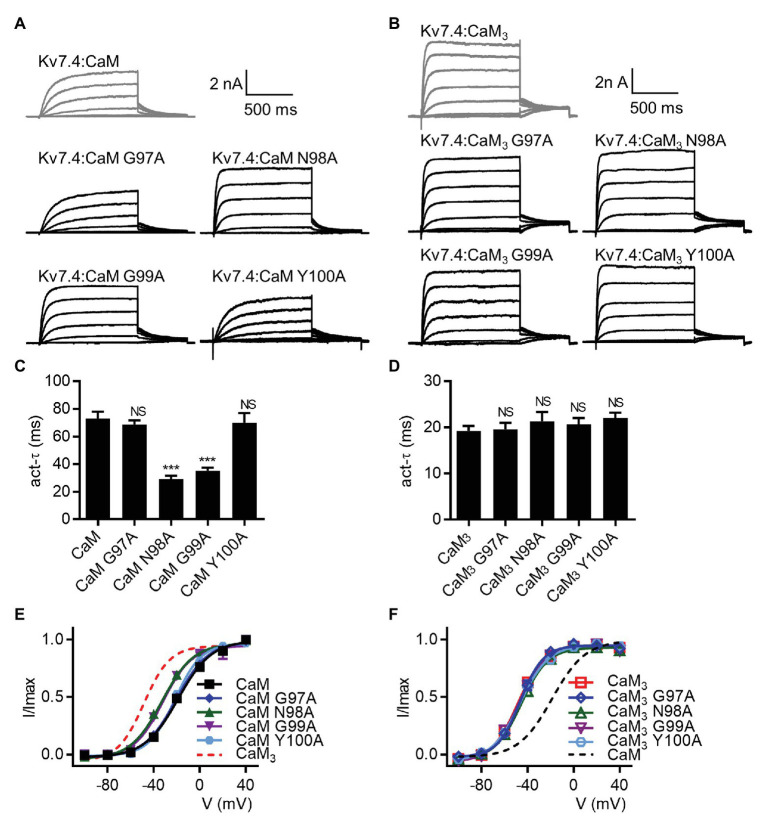
CaM mutations N98A and G99A affect the regulation of Kv7.4 activation. **(A)** Representative current traces of cells co-expressing Kv7.4 with CaM-based mutants. **(B)** Representative current traces of cells co-expressing Kv7.4 with CaM_3_-based mutants. **(C)** Average time constants at 40 mV for the fast activation component of Kv7.4:CaM (*N* = 13), Kv7.4:CaM G97A (*N* = 15), Kv7.4:CaM N98A (*N* = 12), Kv7.4:CaM G99A (*N* = 10), and Kv7.4:CaM Y100A (*N* = 17). **(D)** Average time constants at 40 mV for the fast activation component of Kv7.4:CaM_3_ (*N* = 11), Kv7.4:CaM_3_ G97A (*N* = 11), Kv7.4:CaM_3_ N98A (*N* = 11), Kv7.4:CaM_3_ G99A (*N* = 14), and Kv7.4:CaM_3_ Y100A (*N* = 9). **(E)** Voltage-dependent activation curves for Kv7.4:CaM (*N* = 13), Kv7.4:CaM G97A (*N* = 15), Kv7.4:CaM N98A (*N* = 12), Kv7.4:CaM G99A (*N* = 10), and Kv7.4:CaM Y100A (*N* = 17). **(F)** Voltage-dependent activation curves for Kv7.4:CaM_3_ (*N* = 11), Kv7.4:CaM_3_ G97A (*N* = 11), Kv7.4:CaM_3_ N98A (*N* = 11), Kv7.4:CaM_3_ G99A (*N* = 14), and Kv7.4:CaM_3_ Y100A (*N* = 9). Error bars show SEM. *N* indicates the number of cells. Asterisks indicate significance: ^***^p < 0.001. *NS* indicates not significant.

Sun and MacKinnon (2017) have previously shown that four residues in the CaM EF3 (Gly97, Asn98, Gly99, and Tyr100) are involved in the newly discovered interface between CaM and the S2–S3 loop of Kv7.1 channels (Supplementary Figure S9). We generated CaM mutants in which Gly97, Asn98, Gly99, or Tyr100 were mutated to an alanine based on WT CaM (CaM G97A, CaM N98A, CaM G99A, and CaM Y100A) or CaM_3_ (CaM_3_ G97A, CaM_3_ N98A, CaM_3_ G99A, and CaM_3_ Y100A). Kv7.4 channels co-expressed with all these CaM mutants yielded functional currents (Figures 7A,B). Compared with WT CaM, CaM N98A and CaM G99A accelerated the activation rate (Figure 7C; *τ* values at 40 mV: Kv7.4:CaM = 73.1 ± 4.8 ms, Kv7.4:CaM N98A = 29.1 ± 2.5 ms, and Kv7.4:CaM G99A = 35.2 ± 2.3 ms) and shifted the voltage-dependent activation curve leftwards (Figure 7E; V_1/2_ values: Kv7.4:CaM = −18.7 ± 1.0 mV, Kv7.4:CaM N98A = −32.8 ± 0.9 ms, and Kv7.4:CaM G99A = −31.9 ± 1.8 ms). Kv7.4 co-expressed with CaM G97A or CaM Y100A generated activation properties identical to Kv7.4 co-expressed with WT CaM in terms of both the activation rate (Figure 7C; *τ* values at 40 mV: Kv7.4:CaM G97A = 68.6 ± 3.1 ms and Kv7.4:CaM Y100A = 70.0 ± 7.0 ms) and the voltage dependence of activation (Figure 7E; V_1/2_ values: Kv7.4:CaM N98A = −20.4 ± 1.0 ms and Kv7.4:CaM G99A = −21.6 ± 0.8 ms), suggesting that the two residues of CaM were not involved in the regulation of Kv7.4 activation. All mutants based on CaM_3_ (CaM_3_ G97A, CaM_3_ N98A, CaM_3_ G99A, and CaM_3_ Y100A) phenocopied the effects of CaM_3_ on the activation rate (Figure 7D; *τ* values at 40 mV: Kv7.4:CaM_3_ = 19.2 ± 1.1 ms, Kv7.4:CaM_3_ G97A = 19.5 ± 1.4 ms, Kv7.4:CaM_3_ N98A = 21.3 ± 2.0 ms, Kv7.4:CaM_3_ G99A = 20.7 ± 1.3 ms, and Kv7.4:CaM_3_ Y100A = 22.1 ± 1.1 ms) and the voltage dependence of activation (Figure 7F; V_1/2_ values: Kv7.4:CaM_3_ = −48.4 ± 1.0 mV, Kv7.4:CaM_3_ G97A = −46.3 ± 1.2 mV, Kv7.4:CaM_3_ N98A = −45.4 ± 1.3 mV, Kv7.4:CaM_3_ G99A = −48.1 ± 1.1 mV, and Kv7.4:CaM_3_ Y100A = −48.0 ± 0.9 mV). Taken together, the effects of the two mutations N98A and G99A in the CaM EF3 were similar to the effects of the five mutations (C156A, C157A, C158V, R159A, and R161A) in the Kv7.4 S2–S3 loop, which potentially interacted with the CaM EF3, providing further supporting evidence for the model in which CaM modulated Kv7.4 activation through the S2-S3 loop.

### CaM Regulates Activation of Kv7.4 Isoform b

**Figure 8:**
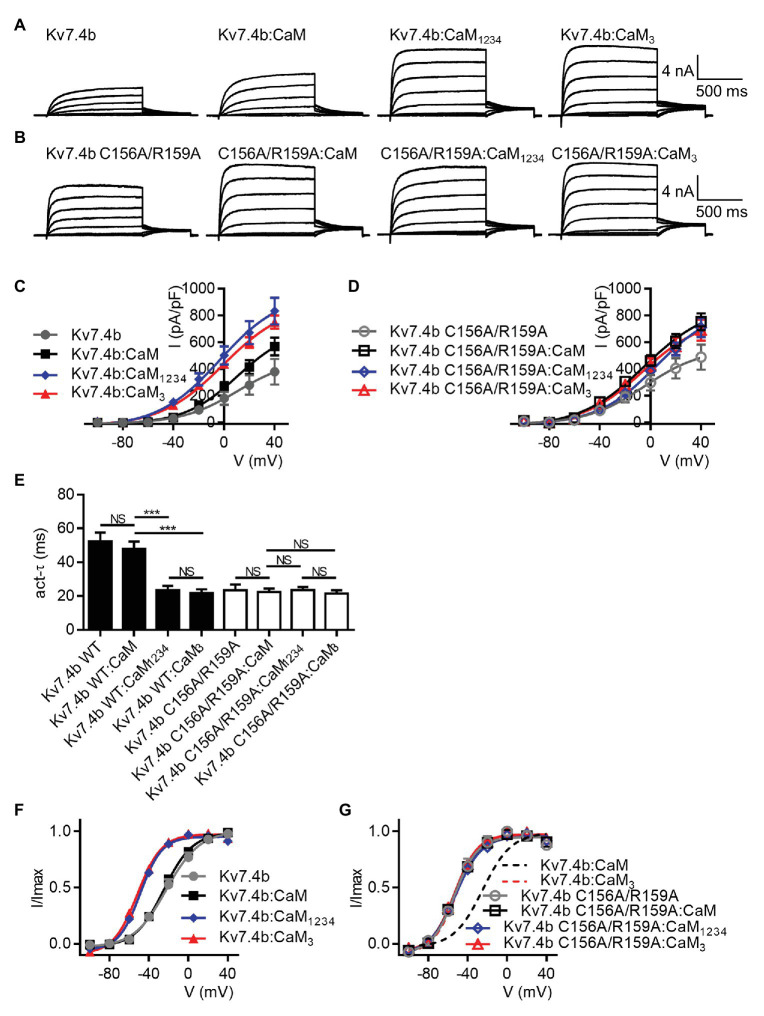
CaM regulates activation of Kv7.4 isoform b. **(A)** Representative current traces of Kv7.4b without overexpression of exogenous CaM and Kv7.4b with overexpression of exogenous CaM, CaM_1234_, or CaM_3_. **(B)** Representative current traces of Kv7.4b C156A/R159A without overexpression of exogenous CaM and Kv7.4b C156A/R159A with overexpression of exogenous CaM, CaM_1234_, or CaM_3_. **(C)** Corresponding current density-voltage relations of Kv7.4b (*N* = 7), Kv7.4b:CaM (*N* = 10), Kv7.4b:CaM_1234_ (*N* = 9), and Kv7.4b:CaM_3_ (*N* = 10). **(D)** Corresponding current density-voltage relations of Kv7.4b C156A/R159A (*N* = 7), Kv7.4b C156A/R159A:CaM (*N* = 9), Kv7.4b C156A/R159A:CaM_1234_ (*N* = 8), and Kv7.4b C156A/R159A:CaM_3_ (*N* = 8). **(E)** Average time constants at 40 mV for the fast activation component of Kv7.4b (*N* = 7), Kv7.4b:CaM (*N* = 10), Kv7.4b:CaM_1234_ (*N* = 9), Kv7.4b:CaM_3_ (*N* = 10), Kv7.4b C156A/R159A (*N* = 7), Kv7.4b C156A/R159A:CaM (*N* = 9), Kv7.4b C156A/R159A:CaM_1234_ (*N* = 8), and Kv7.4b C156A/R159A:CaM_3_ (*N* = 8). **(F)** Voltage-dependent activation curves for Kv7.4b (*N* = 7), Kv7.4b:CaM (*N* = 10), Kv7.4b:CaM_1234_ (*N* = 9), and Kv7.4b:CaM_3_ (*N* = 10). **(G)** Voltage-dependent activation curves for Kv7.4b C156A/R159A (*N* = 7), Kv7.4b C156A/R159A:CaM (*N* = 9), Kv7.4b C156A/R159A:CaM_1234_ (*N* = 8), and Kv7.4b C156A/R159A:CaM_3_ (*N* = 8). Error bars show SEM. *N* indicates the number of cells. Asterisks indicate significance: ^***^p < 0.001. *NS* indicates not significant.

There are two alternatively spliced isoforms of human Kv7.4, called Kv7.4 isoform a and Kv7.4 isoform b. Most studies including ours, used Kv7.4 isoform a, which we and others called Kv7.4. To further the understanding of CaM regulation of both the two isoforms, we also performed experiments on Kv7.4 isoform b, which we called Kv7.4b to distinguish from isoform a. Whole-cell recordings were performed on CHO cells transfected with Kv7.4b alone or together with WT CaM, CaM_1234_, or CaM_3_. Kv7.4b with only endogenous CaM and Kv7.4b with overexpression of exogenous WT CaM showed the same activation rate (*τ* values at 40 mV: Kv7.4b = 52.5 ± 5.0 ms; Kv7.4b:CaM = 48.0 ± 4.2 ms) and voltage dependence of activation (V_1/2_ values: Kv7.4b = −22.2 ± 2.0 mV; Kv7.4b:CaM = −24.9 ± 1.0 mV). Overexpression of CaM_1234_ significantly accelerated the activation rate (*τ* value at 40 mV: Kv7.4b:CaM_1234_ = 23.4 ± 2.7 ms) and shifted the voltage dependent activation leftwards markedly (V_1/2_ value: Kv7.4b:CaM_1234_ = −48.8 ± 1.1 mV). CaM_3_ mirrored the facilitating effects of CaM_1234_ on both activation kinetics (*τ* value at 40 mV: Kv7.4b:CaM_3_ = 21.8 ± 2.2 ms) and voltage-dependence activation (V_1/2_ value: Kv7.4b:CaM_3_ = −51.6 ± 1.1 mV). To probe effects of the Kv7.4b S2–S3 loop on CaM regulation, we also performed whole-cell recordings on CHO cells transfected with Kv7.4b C156A/R159A alone or together with WT CaM, CaM_1234_, or CaM_3_. According to the statistical results, there was no significant difference among the four groups in both the activation rate (*τ* values at 40 mV: Kv7.4b C156A/R156A = 23.5 ± 3.4 ms; Kv7.4b C156A/R159A:CaM = 22.5 ± 2.0 ms; Kv7.4b C156A/R159A:CaM_1234_ = 23.6 ± 1.8 ms; Kv7.4b C156A/R159A:CaM_3_ = 21.6 ± 1.9 ms) and the voltage dependence of activation (V_1/2_ values: Kv7.4b C156A/R156A = −53.2 ± 2.0 mV; Kv7.4b C156A/R159A:CaM = −53.5 ± 0.9 mV; Kv7.4b C156A/R159A:CaM_1234_ = −52.4 ± 1.3 mV; Kv7.4b C156A/R159A:CaM_3_ = −53.3 ± 0.9 mV), suggesting that the double mutation C156A/R159A in the S2–S3 loop also abolished CaM regulation of Kv7.4b activation. Taken together, our work revealed a unified mechanism for CaM regulation of both isoforms of Kv7.4 channels (Figure 8).

## Discussion

With the properties of opening at subthreshold membrane potentials and lacking inactivation, Kv7 channels hold a pivotal position in controlling excitability in the heart and the nervous system (Barhanin et al., 1996; Sanguinetti et al., 1996; Wang et al., 1996, 1998; Jentsch, 2000; Delmas and Brown, 2005; Hernandez et al., 2008; Brown and Passmore, 2009). CaM conferred effective regulation to Kv7 channels. Though the importance of CaM regulation of Kv7 channels has been recognized, the regulation mechanism remains elusive. Earlier studies mostly focused on the role of the C-terminal domain of Kv7 channels in CaM binding and CaM regulation. Mutations in the C-terminal domain impairing CaM binding affect channel assembly, trafficking, and gating (Ghosh et al., 2006; Shamgar et al., 2006; Etxeberria et al., 2008; Alaimo et al., 2009, 2018; Liu and Devaux, 2014; Tobelaim et al., 2017; Chang et al., 2018). Here, our data provided new insight into CaM regulation of Kv7 channels by proving that the S2–S3 loop of Kv7.4 channels is essential for CaM-mediated calcium-dependent regulation of Kv7.4 activation.

Previous studies have shown that CaM conferred intracellular calcium signals to Kv7 channels (Gamper and Shapiro, 2003; Gamper et al., 2005; Sihn et al., 2016; Gomis-Perez et al., 2017; Tobelaim et al., 2017; Chang et al., 2018). To probe the calcium-dependent regulation of Kv7.4 channels mediated by CaM, we performed electrophysiology experiments on CHO cells transfected with Kv7.4 anole or together with WT CaM or CaM mutants with impaired calcium binding abilities. CHO cells have endogenous CaM, which is important for many cellular physiological activities, and thus cannot be knocked out. Overexpression of exogenous CaM or CaM mutants could knockdown the effects of endogenous CaM, which is a common method used by most of the previous studies to probe calcium-dependent CaM regulation. Compared with Kv7.4 channels with only endogenous CaM of CHO cells, overexpression of exogenous WT CaM increased current amplitudes over 2-fold but did not change activation properties, and overexpression of exogenous CaM_1234_ increased current amplitudes about 5-fold and facilitated channel activation as well. CaM_1234_ sharply accelerated the activation rate and produced a large leftward shift in the voltage-dependent activation, similar to the effects of CaM_1234_ on Kv7.2 (Gomis-Perez et al., 2017), Kv7.3 (Gomis-Perez et al., 2017), and Kv7.5 (Chang et al., 2018) and implying a unified mechanism in which Apo/CaM facilitates Kv7.2–7.5 activation, or in other words, in which Ca^2+^/CaM inhibits Kv7.2–7.5 activation.

We also measured surface expression of Kv7.4 with only endogenous CaM and Kv7.4 with overexpression of exogenous WT CaM and CaM_1234_ to further explain the different current amplitudes. Kv7.4 co-expressed with WT CaM and Kv7.4 co-expressed with CaM_3_ showed similar surface expression and about 3.5-fold the surface expression of Kv7.4 with only endogenous CaM, suggesting that CaM enhances Kv7.4 surface expression in a calcium-independent manner. We also measured Kv7.4 total expression of the three groups, and the total expression of Kv7.4 with overexpression of exogenous WT CaM or CaM_1234_ was about twice the total expression of Kv7.4 channels with only endogenous CaM (Supplementary Figures S4A,B). Taken together, increased surface expression accounted for the greater current amplitudes of Kv7.4 with overexpression of exogenous WT CaM than Kv7.4 with only endogenous CaM, and Kv7.4 with overexpression of exogenous CaM_1234_ generated further greater current amplitudes due to both facilitated activation and increased surface expression. As the facilitating effect of the overexpression of exogenous WT CaM or CaM_1234_ on Kv7.4 surface expression is more significant than the facilitating effect on Kv7.4 total expression, we deem that the increase of Kv7.4 surface expression is likely due to not only the increase of Kv7.4 total expression but also enhanced channel trafficking.

Of note, Kv7.4 with overexpression of exogenous WT CaM showed activation properties equivalent to Kv7.4 with only endogenous CaM, possibly because exogenous WT CaM is essentially the same as endogenous CaM except the much higher expression level. CaM was reported to be an auxiliary subunit of Kv7 channels (Wen and Levitan, 2002). Disrupted CaM interactions with Kv7 channels have been shown to be tightly associated with severely impaired Kv7 surface expression (Ghosh et al., 2006; Shamgar et al., 2006; Etxeberria et al., 2008; Alaimo et al., 2009, 2018; Liu and Devaux, 2014; Chang et al., 2018), illustrating the vital role of CaM in Kv7 assembly and trafficking, which is also supported by our study. Additionally, the stoichiometry for CaM binding to the Kv7 subunit was 1:1 (Sun and MacKinnon, 2017), and Bernardo-Seisdedos et al. (2018) reported that the Kv7.2 A and B helices could not be purified in the absence of CaM and that the complex could not be dissociated at any calcium concentration *in vitro*. Therefore, we deem that there would be CaM strongly and stably binding to the functional plasma membrane Kv7.4 channels, which has been properly assembled and transported, at a stoichiometry of 1:1, although more Kv7.4 channels was expressed on the cell surface due to the overexpression of exogenous WT CaM. However, the already bound CaM controls the regulating activation of the plasma membrane Kv7.4 channels, so the different expression levels of endogenous CaM and overexpressed exogenous WT CaM did not change Kv7.4 activation.

We also studied the effects CaM_12_, CaM_34_, CaM_3_, and CaM_4_ on Kv7.4 activation to probe the role of each lobe or EF hand in regulating Kv7.4 channels. CaM_34_ and CaM_3_ caused facilitating effects equivalent to CaM_1234_ on Kv7.4 activation kinetics and the voltage dependence of activation, supporting a key role of the EF3 in CaM regulation of Kv7.4 activation. CaM_12_ did not mirror the effects of WT CaM of Kv7.4 activation, possibly because calcium binding could enhance the binding of the N-lobe to the B helix of Kv7.4 (Chang et al., 2018), and such configuration changes might have slight effects on channel function. However, the changes of Kv7.4 activation caused by CaM_12_ were much slighter than the changes caused by CaM_1234_, CaM_34_, and CaM_3_. Our data largely match the results by Chang et al. (2018), but there is a difference in the effects of CaM_4_ on Kv7.4 activation. We found that CaM_4_ did not change Kv7.4 activation kinetics or the voltage dependence of activation, whereas Chang et al. (2018) showed that CaM_4_ facilitated Kv7.4 activation though the facilitating effects is much weaker than CaM_3_, CaM_34_, and CaM_1234_. This difference might result from the different experimental approaches, in which Chang et al. (2018) used perforated-patch configuration to test cells under natural resting calcium levels but we used whole-cell configuration to substitute the cell cytosolic composition by the pipette solution. Moreover, activation of Kv7.4 co-expressed with WT CaM was inhibited by increasing intracellular calcium levels, while CaM_1234_ and CaM_3_ rendered Kv7.4 insensitive to the changing calcium concentrations, further emphasizing the role of calcium in CaM regulation of Kv7.4 channels and implying that CaM_1234_ and CaM_3_ facilitate Kv7.4 activation essentially by abolishing the calcium-dependent inhibition.

The free calcium concentration in the regular pipette solution is 80 nM, which is within the basal cytosolic calcium concentrations (10–100 nM). Free CaM exists in its Apo-forms in the basal cytosolic calcium concentrations [K_d_(C-lobe) = 3.4 μM, K_d_(N-lobe) = 14 μM; Linse et al., 1991]. However, CaM binding to some target peptides or proteins can dramatically increase calcium affinities (Olwin et al., 1984; Olwin and Storm, 1985; Peersen et al., 1997; Black et al., 2005; Evans and Shea, 2009; Findeisen et al., 2013). For instance, the calcium affinities of CaM binding to the IQ motif of Cav channels, kinase skMLCK or kinase MKII are quite high with Kd values much lower than 100 nM (Peersen et al., 1997; Black et al., 2005). In this study, the calcium chelators, EGTA and BAPTA-AM exerted effects on Kv7.4 activation in the same facilitating direction as CaM_1234_, CaM_12_, CaM_34_, and CaM_3_, suggesting that there are some tonic occupations by calcium in the EF hands under the basal intracellular calcium level. Previous studies have also reported changed Kv7 currents by decreasing intracellular calcium concentrations from the basal level (Ghosh et al., 2006; Shamgar et al., 2006; Chang et al., 2018). These observations are possibly due to increased calcium affinities of CaM when binding to Kv7 channels.

Structural studies have reported that the CaM EF3 interacted with the Kv7.1 S2–S3 loop, which is conserved among Kv7 isoforms (Sun and MacKinnon, 2017). To explore if the important functional role of the CaM EF3 is associated with the potential interaction between the CaM EF3 and the Kv7.4 S2–S3, we mutated amino acids either in the Kv7.4 S2–S3 loop or in the CaM EF3 to a nonpolar amino acid. Five mutations (C156A, C157A, C158V, R159A, and R161A) in the Kv7.4 S2–S3 loop and two mutations (N98A and G99A) in the CaM EF3 led to decreased inhibitory effects of Ca^2+^/CaM, facilitating Kv7.4:CaM activation in terms of both the activation rate and the voltage dependence of activation. The double mutation C156A/R159A within the S2–S3 loop completely abolished the inhibitory effects of Ca^2+^/CaM on Kv7.4 activation and rendered Kv7.4 insensitive to changing intracellular calcium levels, which further emphasizes the importance of the Kv7.4 S2–S3 loop in CaM regulation. We also performed co-immunoprecipitation experiments to probe the effect of C156A/R159A on CaM binding. Compared with WT Kv7.4, the double mutant C156A/R159A showed weaker association with Ca^2+^/CaM but unchanged association with CaM_3_ and Apo/CaM, suggesting that the Kv7.4 S2–S3 loop interacts with the Ca^2+^/EF3 but has no association with the Apo/EF3. The co-immunoprecipitation results coincide with our electrophysiological results. Mutations in the Kv7.4 S2–S3 loop decreased Ca^2+^/CaM binding as well as Ca^2+^/CaM inhibition on channel activation, while these mutations had no effect on CaM_3_ binding as well as activation of Kv7.4 co-expressed with CaM_3_, suggesting that Ca^2+^/CaM inhibited Kv7.4 activation through an interaction with the Kv7.4 S2–S3 loop.

We also probed CaM regulation of Kv7.4 isoform b, activation of which was reported by Sihn et al. (2016) to be not regulated by CaM. Our results are strikingly different from Sihn et al. (2016) and show that CaM regulates Kv7.4b activation in a calcium-dependent manner similarly to the regulation of Kv7.4 isoform a. CaM_1234_ facilitated Kv7.4b activation in terms of both activation kinetics and the voltage dependence of activation, and CaM_3_ phenocopied the facilitating effects of CaM_1234_. The double mutation C156A/R159A abolished CaM regulation of Kv7.4b activation. These results emphasized the crucial roles of the EF3 and the S2–S3 loop in CaM regulating activation of Kv7.4b. There are cases where the earlier studies failed to find CaM regulation of Kv7.1 and Kv7.3 (Gamper et al., 2005), both of which were later shown to be regulated by CaM (Sachyani et al., 2014; Gomis-Perez et al., 2017; Tobelaim et al., 2017; Chang et al., 2018). Our work proved a unified rather than isoform-dependent mechanism for CaM regulation of Kv7.4 channels.

**Figure 9:**
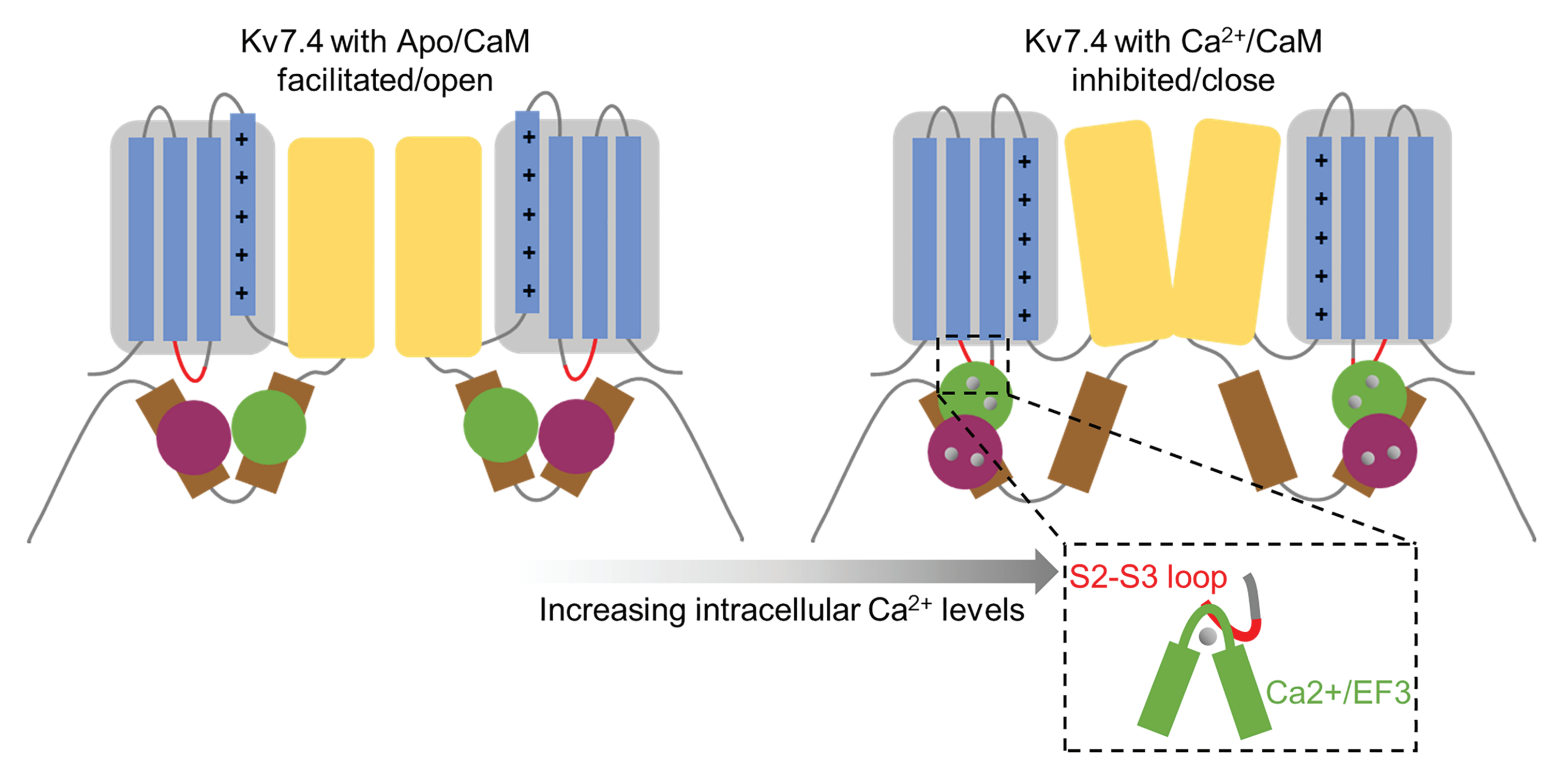
A model for CaM-mediated calcium-dependent regulation of Kv7.4 activation. The gray box represents the voltage sensor domain containing the Kv7.4 transmembrane helixes S1–S4, with positively charged amino acids (shown as plus signs) in the S4 helix. The S2–S3 loop is shown as a red curve. The yellow box represents the pore domain. The brown rectangles represent the A and B helices. The CaM N-lobe is colored in green, and the C-lobe is colored in purple. The gray circles represent calcium bound to CaM. Apo/CaM binds to Kv7.4 channels with the Apo/N-lobe interacting with the B helix and the Apo/C-lobe interacting with the A helix, and Apo/CaM has no association with the Kv7.4 S2–S3 loop. Upon calcium loading, the Ca^2+^/N-lobe remains anchored on the B helix, while the Ca^2+^/C-lobe releases interaction with the A helix and binds to the B helix through a much smaller interaction surface, making it possible for the Ca^2+^/EF3 to contact the Kv7.4 S2–S3 loop and affect motivation of the voltage sensor and opening of the coupled pore.

To the best of our knowledge, the mechanism for CaM-mediated calcium-dependent regulation of voltage-gated channels has remained puzzling since the CaM binding sites were initially found. Heretofore, the most-studied structural presentation accounting for the mechanism for CaM regulation of channel activation comes from SK4 channels (Lee and MacKinnon, 2018). It was proposed that Apo/CaM binds to SK4 channels through the interaction of Apo/C-lobe with the SK4 A and B helices, and calcium loading allows the Ca^2+^/N-lobe to remain anchored on the A and B helices and enables the Ca^2+^/N-lobe to contact the S4–S5 linker of SK4 channels and further affect pore opening. In Kv7 channels, it has been widely assumed that the Ca^2+^-dependent CaM regulation is also driven by conformational changes derived from calcium loading, but detailed interpretation remained unresolved. Apo/CaM binds to Kv7 channels in a clamped form, in which the C-lobe interacts with the A helix of Kv7 channels and the N-lobe interacts with the B helix (Bernardo-Seisdedos et al., 2018; Chang et al., 2018). Calcium binding allow the Ca^2+^/N-lobe to stay anchored on the Kv7 B helix in a similar configuration to the apo/N-lobe (Sachyani et al., 2014; Strulovich et al., 2016; Sun and MacKinnon, 2017; Bernardo-Seisdedos et al., 2018; Chang et al., 2018), while there are striking differences between the Ca^2+^/C-lobe and Apo/C-lobe conformations. Structural studies of the interaction between CaM and the Kv7.4 A and B helices showed that calcium binding makes the C-lobe lose interaction with the A helix and bind to the B helix weakly through a much smaller interaction surface (Xu et al., 2013; Chang et al., 2018). Xu et al. (2013) proposed that the interaction of apo/CaM with the Kv7.4 A helix may affect the neighboring S6 in some way to favor channel opening and, in contrast, when CaM switches to Ca^2+^/CaM and binds to the helix B, loss of the interaction with the A helix causes inhibition of the currents. Our data demonstrate that the third EF hand of CaM and the S2–S3 loop of Kv7.4 channels play crucial roles in CaM-mediated calcium-dependent regulation of Kv7.4 activation, and lead us to propose a new regulation model (Figure 9). Apo/CaM binds only the C-terminal domain of Kv7.4 channels through the interaction of the Ca^2+^/C-lobe with the A helix and the interaction of the Ca^2+^/N-lobe with the B helix, and has no association with the voltage sensor domain of Kv7.4 channels. Thus, the voltage sensor could easily move in response to depolarized membrane potentials, which is coupled to pore opening. Upon calcium loading, the CaM C-lobe releases its interaction with the Kv7.4 A helix and binds to the B helix weakly with a much smaller interaction surface (Xu et al., 2013; Chang et al., 2018), making it possible for the Ca^2+^/EF3 to contact the Kv7.4 S2–S3 loop. This interaction between the Ca^2+^/EF3 and the Kv7.4 S2–S3 loop makes it possible for Ca^2+^/CaM to “pull” the voltage sensor of Kv7.4 channels, and thus offers a force opposing the movement of the voltage sensor in response to depolarized potentials, affecting voltage-dependent pore opening. We could not deny the model proposed by Xu et al. (2013), although our model could better explain the shift in the voltage dependence of activation caused by CaM regulation. Probably, the inhibitory effect of Ca^2+^/CaM on Kv7.4 activation might be a combined effect of the “pulled” voltage sensor and the released helix A. In our model, the interaction of the S2–S3 loop with the Ca^2+^/EF3 rather than the Apo/EF3 is more likely to be driven by calcium-dependent changes of the constitutive interactions between CaM and the Kv7.4 C-terminal domain. However, the residues in the Kv7.4 A and B helices interacting with Apo/CaM and the residues in the Kv7.4 B helix interacting with Ca^2+^/CaM are different from the residues in Kv7.1 to a certain degree, so there might be some differences between Kv7.4 and Kv7.1 in the calcium-dependent interaction with the CaM EF3, accounting for the different direction of CaM regulation.

Limited previous studies explored the role of the Kv7 S2–S3 loop in channel function. Gamper et al. (2006) found that increasing H_2_O_2_ concentrations enhanced Kv7.4 currents and facilitated Kv7.4 activation and that currents of the Kv7.4 triple mutant C156A/C157A/C158A remained unchanged after extracellular application of H_2_O_2_, so they concluded that the triple cysteines in the S2–S3 loop was critical for the oxidative modification. However, their data showed that extracellular application of H_2_O_2_ still facilitated the activation rate of Kv7.4 C156A/C157A/C158A, which seemed contradictory to the unchanged currents. Additionally, before extracellular application of H_2_O_2_ and with only endogenous H_2_O_2_ in cells, Kv7.4 C156A/C157A/C158A showed a faster activation rate and a leftward shift in the half-activation voltage compared with WT Kv7.4, which contrast the facilitating effects of H_2_O_2_ and suggested that the S2–S3 loop might be involved in other regulations that affect channel function as well. Ooi et al. (2013) reported that extracellular application of SNAP that forms nitric oxide (NO) inhibited Kv7.4 currents with an inhibition of about 30%, and that the NO inhibition of the Kv7.4 triple mutant C156A/C157A/C158A was much smaller and almost abolished. They also tested S-nitrosylation of WT Kv7.4 and Kv7.4 C156A/C157A/C158A treated with SNAP and found that Kv7.4 C156A/C157A/C158A showed decreased S-nitrosylation, so they concluded that NO possibly inhibited Kv7.4 currents through S-nitrosylation of the triple cysteines in the S2–S3 loop. Both NO and Ca^2+^/CaM inhibit Kv7.4 channels, and the S2–S3 loop is required for both inhibition, implying a potential correlation between the two regulations. For example, possibly S-nitrosylation of the triple cysteines could favor the interaction between Ca^2+^/CaM and the S2–S3 loop of Kv7.4 channels. Further studies are required to explore the role of the S2-S3 loop in coordinating the process of H_2_O_2_ regulation, NO regulation, and Ca^2+^/CaM regulation.

Overall, our results highlight the importance of the Kv7.4 S2–S3 linker in CaM regulation of channel activation. This is the first study to indicate another site in Kv7.4 channels required for CaM regulation in addition to the C-terminal domain. Furthermore, given that the S2–S3 loop sequence is highly conserved among Kv7.2–7.5, this loop might play a similar role in CaM regulation of all these isoforms. More studies about the S2–S3 loop of the other Kv7 isoforms including Kv7.1 may further our understanding of a unified mechanism for CaM regulation of Kv7 channels.

## Data Availability Statement

The original contributions presented in the study are included in the article/Supplementary Material, further inquiries can be directed to the corresponding author.

## Author Contributions

WZ designed and implemented the experiments, collected and analyzed data, and wrote the paper. ZY supervised the project and provided guidance. Both the authors contributed to the article and approved the submitted version.

### Conflict of Interest

The authors declare that the research was conducted in the absence of any commercial or financial relationships that could be construed as a potential conflict of interest.

## Footnotes

^1^https://somapp.ucdmc.ucdavis.edu/pharmacology/bers/maxchelator/webmaxc/webmaxcS.htm
