# Supplementary figures and images for: The S2–S3 Loop of Kv7.4 Channels Is Essential for Calmodulin Regulation of Channel Activation
Source: Front Physiol. 2021 Jan 20;11:604134. doi: 10.3389/fphys.2020.604134 (PMC7854705; doi:10.3389/fphys.2020.604134)

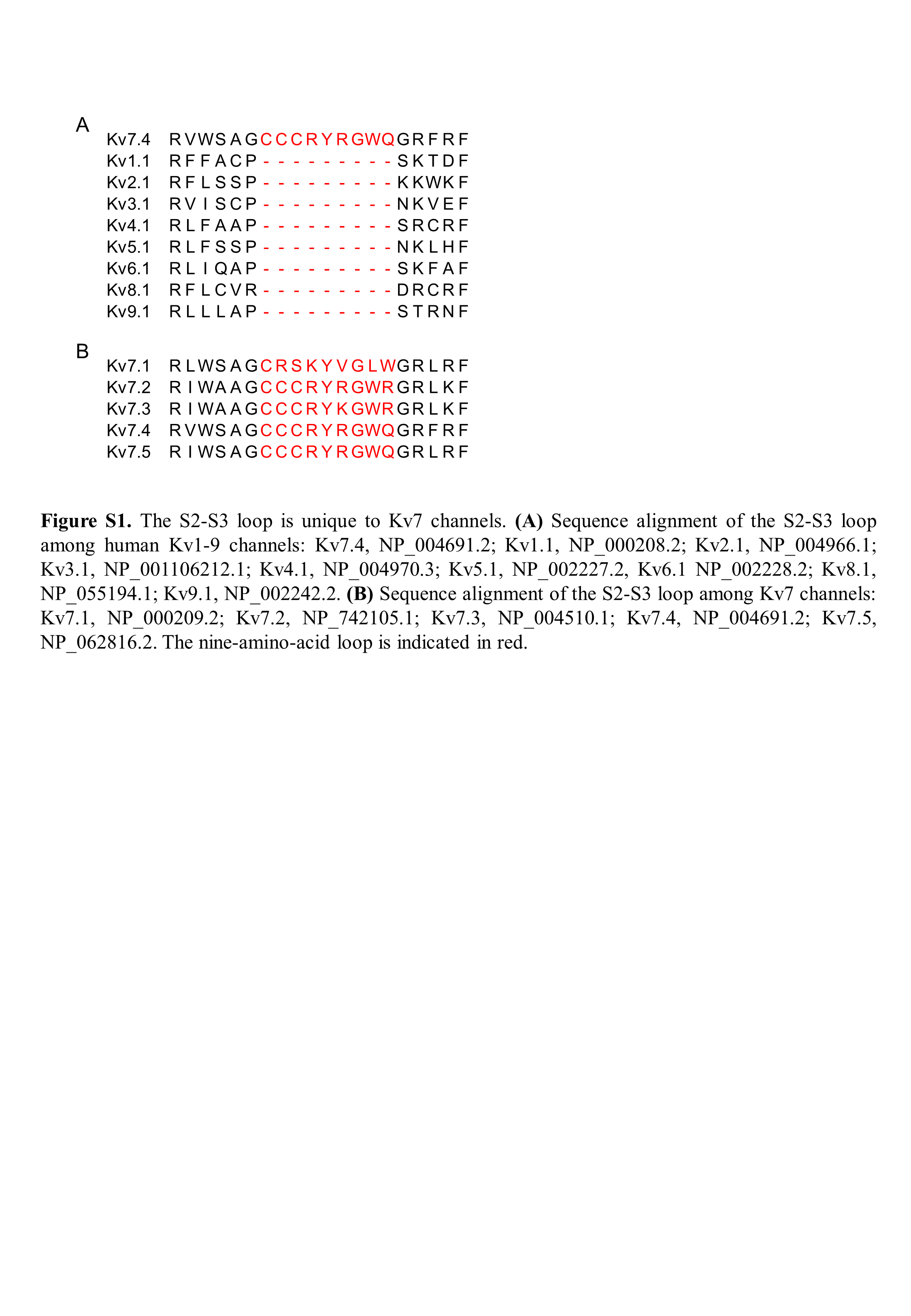

Supplement: Supplementary file 1 [file Image_1.tif]

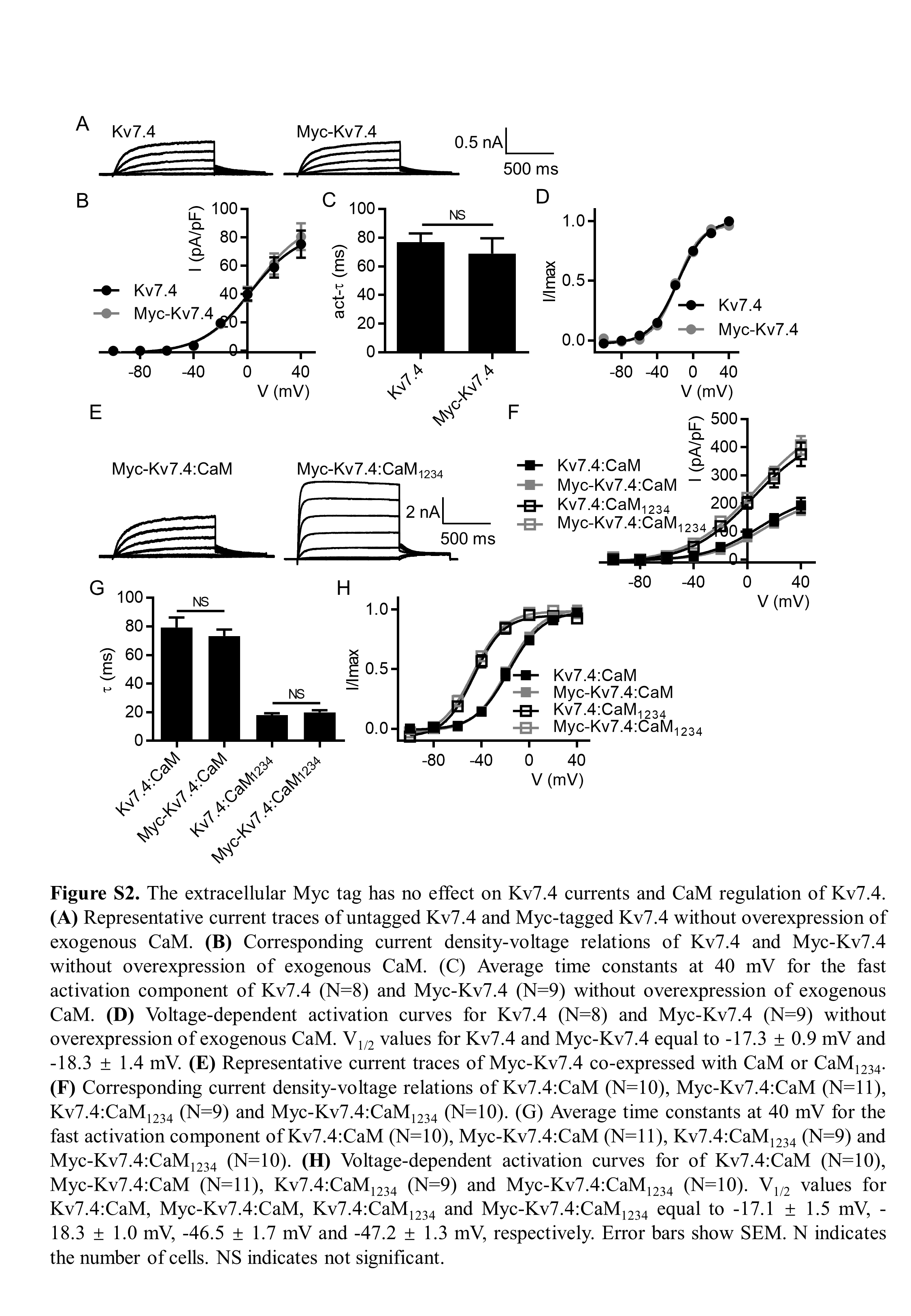

Supplement: Supplementary file 2 [file Image_2.tif]

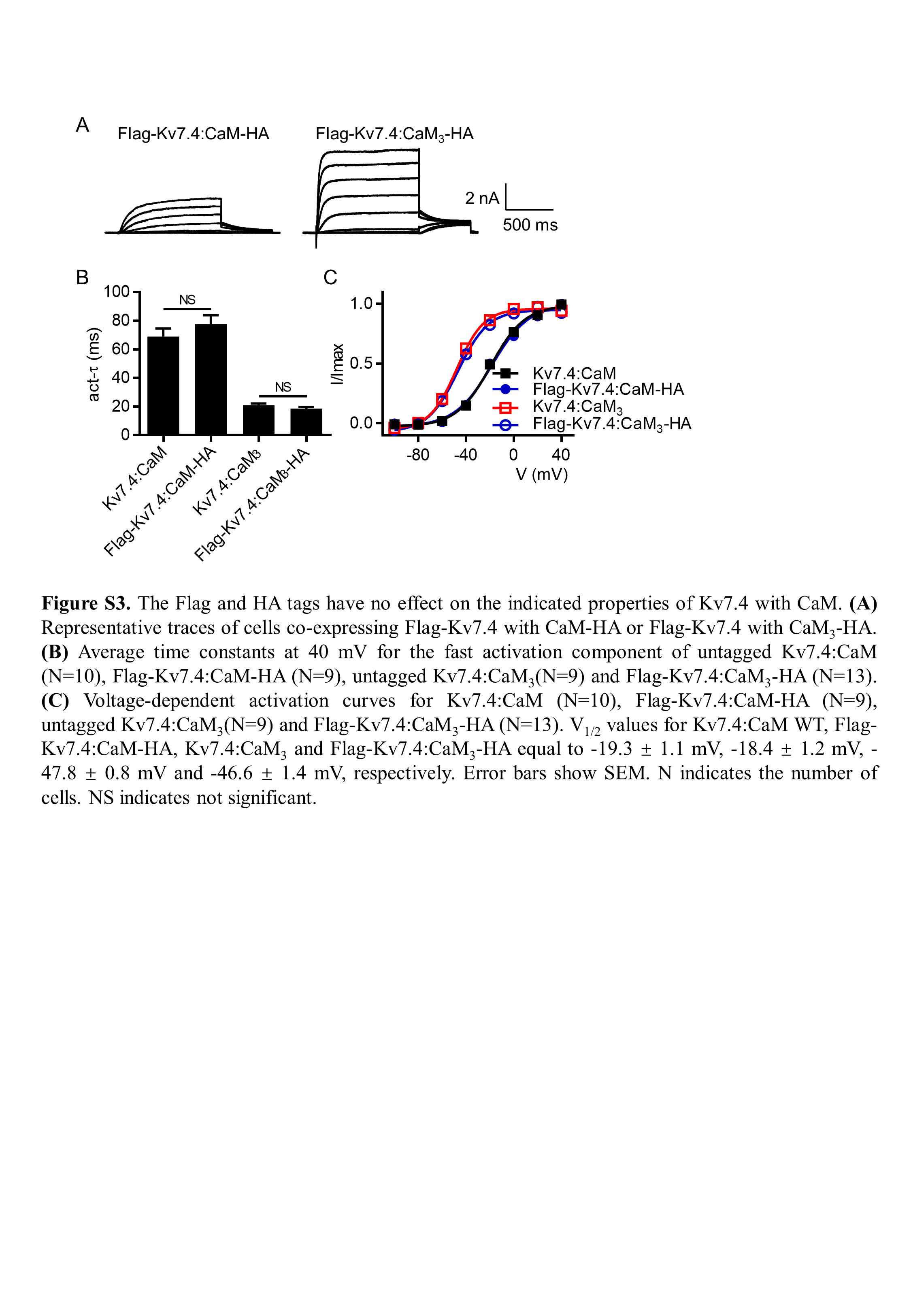

Supplement: Supplementary file 3 [file Image_3.tif]

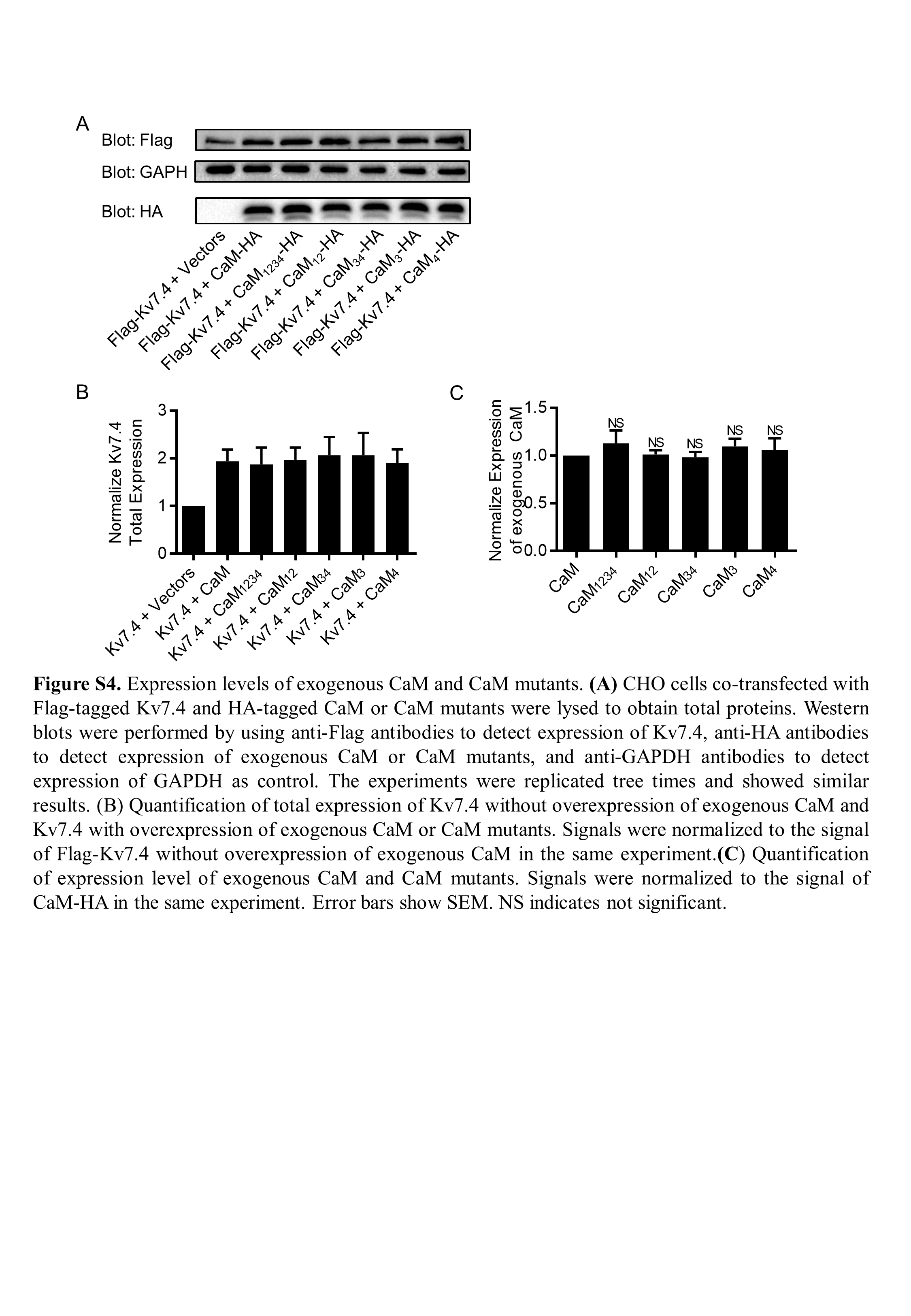

Supplement: Supplementary file 4 [file Image_4.tif]

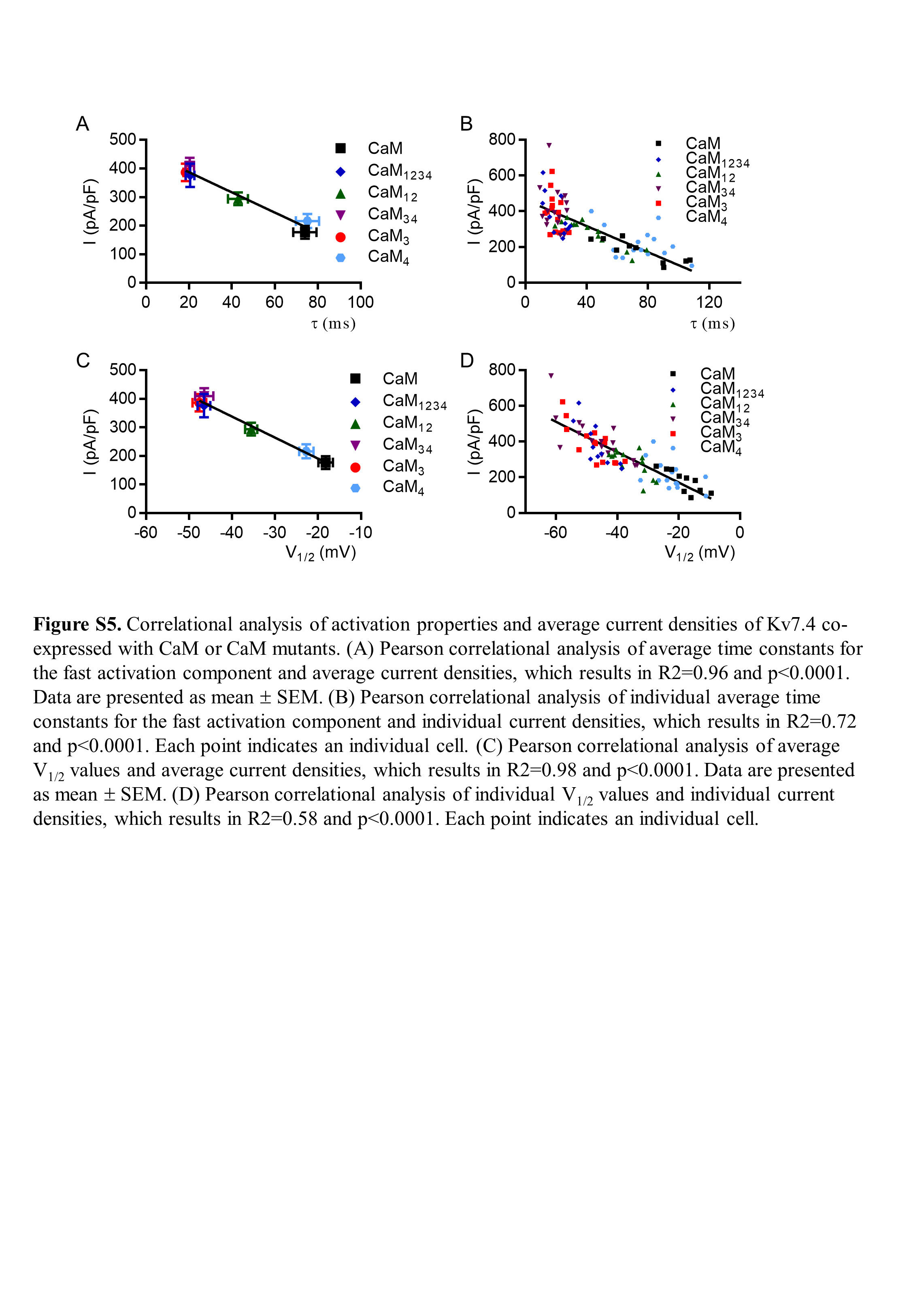

Supplement: Supplementary file 5 [file Image_5.tif]

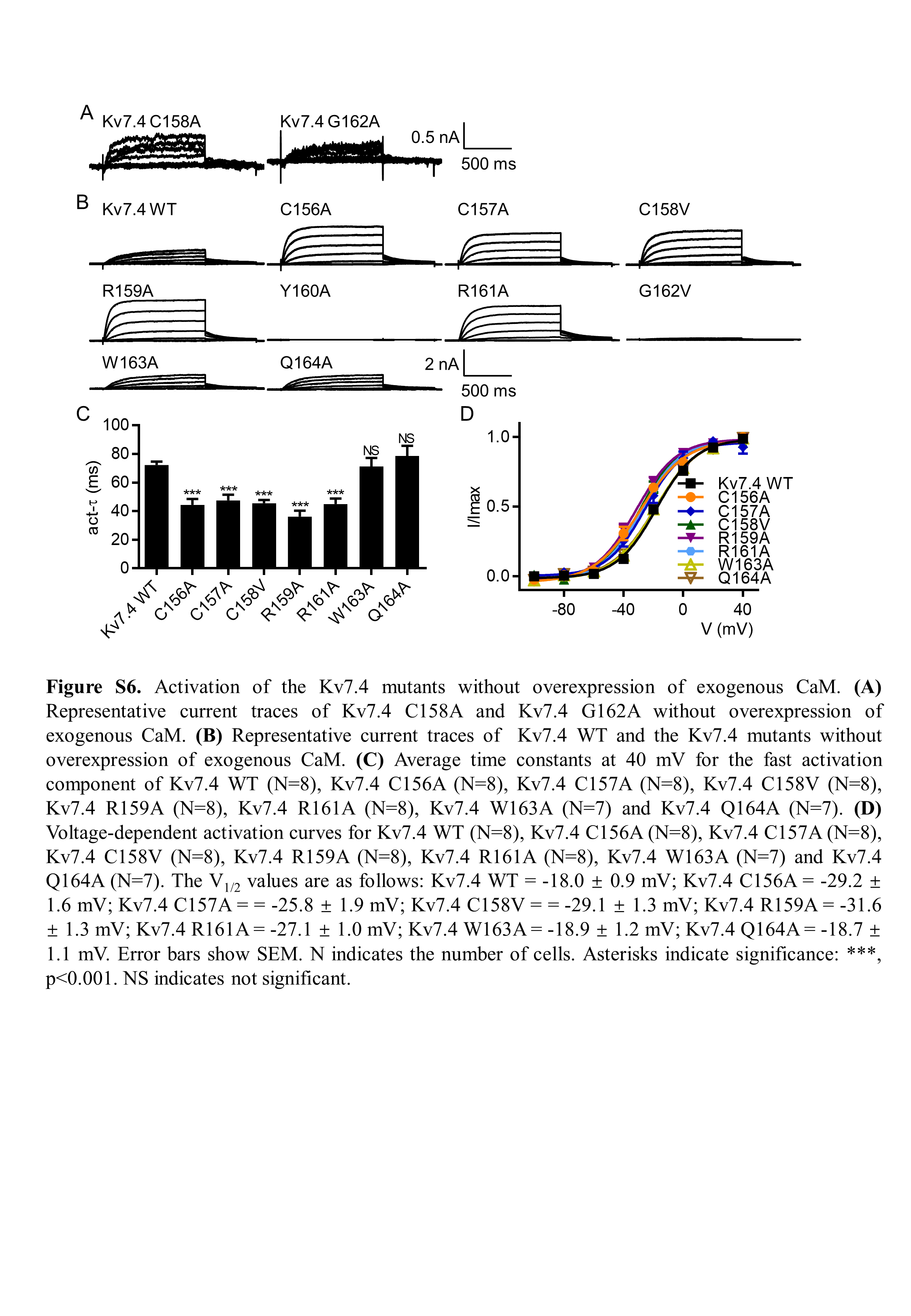

Supplement: Supplementary file 6 [file Image_6.tif]

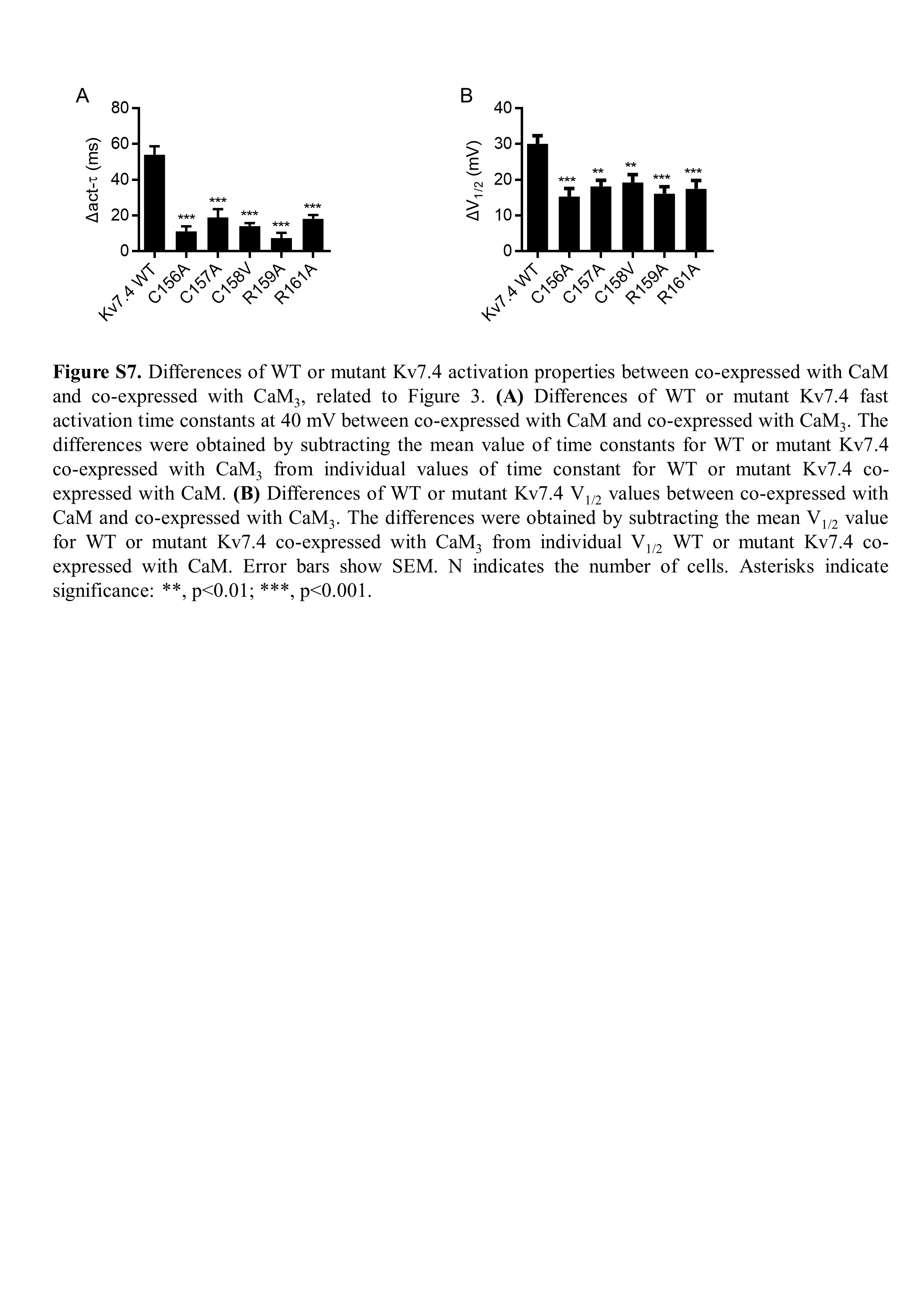

Supplement: Supplementary file 7 [file Image_7.tif]

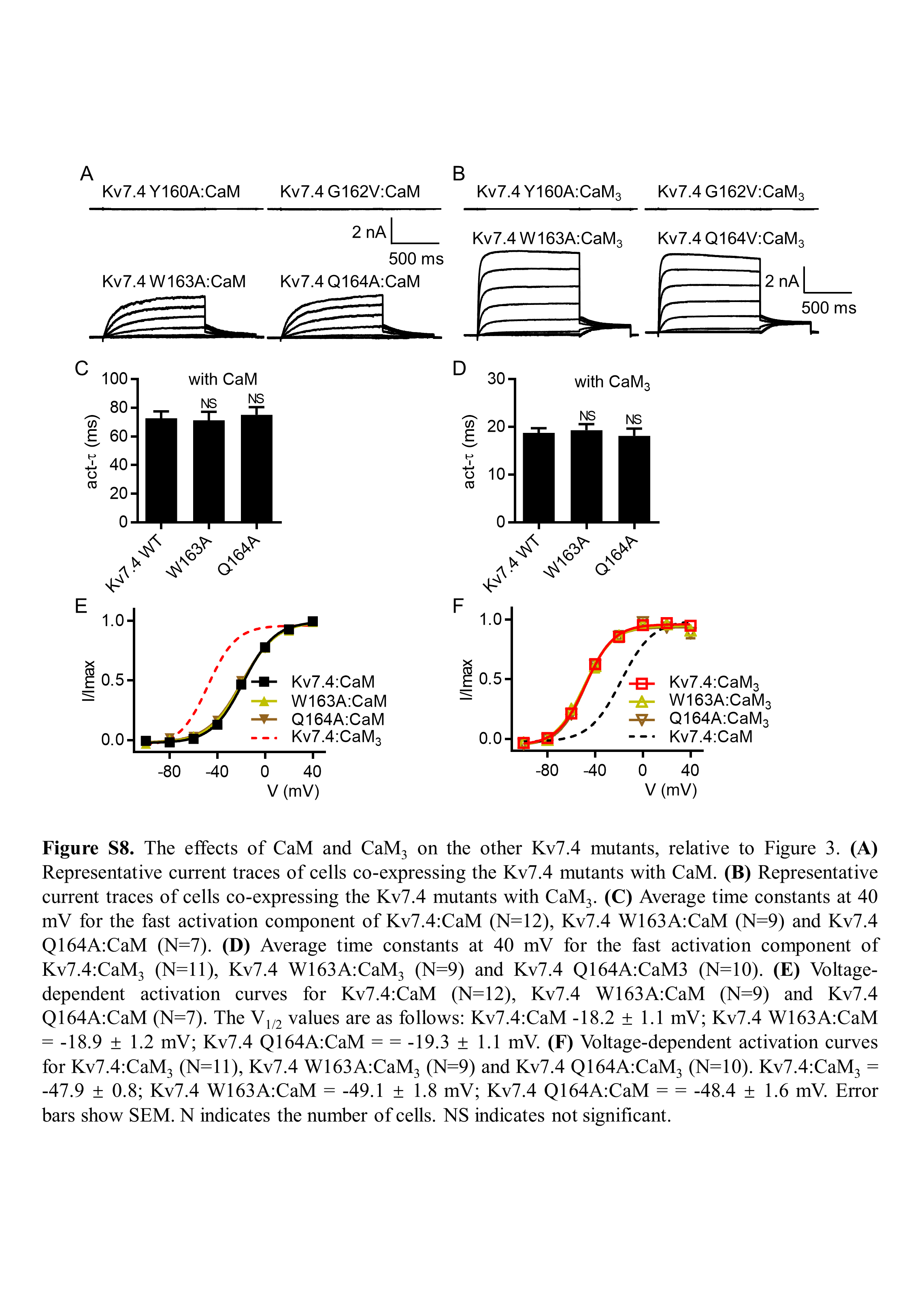

Supplement: Supplementary file 8 [file Image_8.tif]

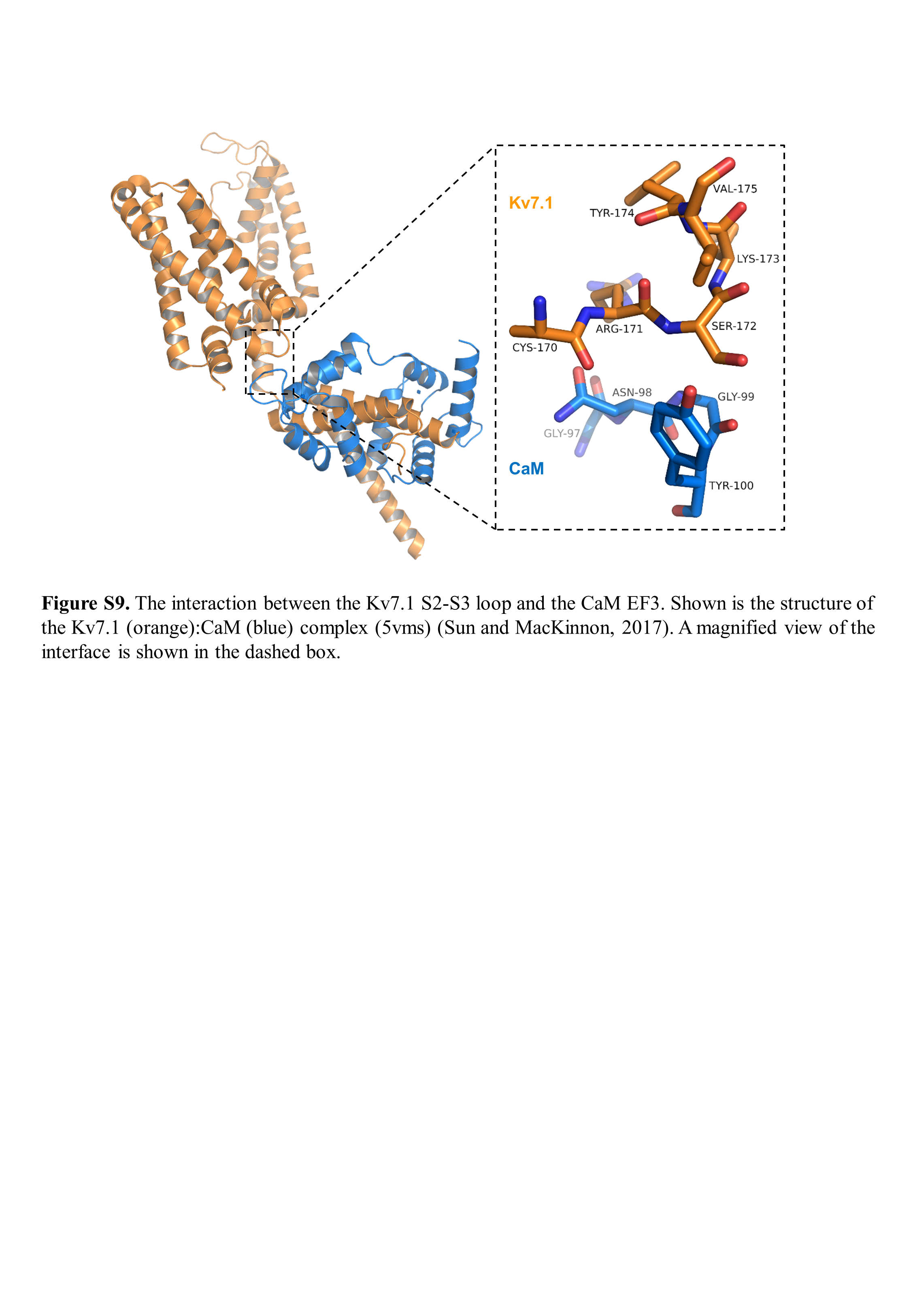

Supplement: Supplementary file 9 [file Image_9.tif]
